# ZDHHC18‐Mediated Palmitoylation of ORF3a Promotes SARS‐CoV‐2 Pathogenesis by Antagonizing TRIM16‐Mediated Ubiquitination and Proteasomal Degradation

**DOI:** 10.1002/advs.75591

**Published:** 2026-05-10

**Authors:** Sidi Yang, Kun Li, Lihong Liu, Linsen Zeng, Qifeng Deng, Jiacheng Huang, Xiaoran Dong, Xin Wang, Jianwei Liang, Hongchao Liu, Hong Peng, Yuxin Lin, Xiaolu Xie, Yuzhen Ye, Tiefeng Xu, Zhaohuan Wang, Chun‐Mei Li, Deyin Guo

**Affiliations:** ^1^ State Key Laboratory of Respiratory Disease National Clinical Research Center for Respiratory Disease Guangzhou Institute of Respiratory Health The First Affiliated Hospital of Guangzhou Medical University Guangzhou P. R. China; ^2^ Guangzhou National Laboratory Guangzhou International Bio‐Island Guangzhou P. R. China; ^3^ Key Laboratory of Tropical Disease Control of Ministry of Education Institute of Human Virology Department of Pathogen Biology and Biosecurity Zhongshan School of Medicine Sun Yat‐Sen University Guangzhou P. R. China; ^4^ MOE Key Laboratory of Gene Function and Regulation State Key Laboratory of Biocontrol School of Life Sciences Sun Yat‐Sen University Guangzhou P. R. China; ^5^ Centre for Infection and Immunity (CII) School of Medicine Shenzhen Campus of Sun Yat‐Sen University Shenzhen P. R. China

**Keywords:** ORF3a, palmitoylation, SARS‐CoV‐2, viral pathogenesis

## Abstract

SARS‐CoV‐2 accessory protein ORF3a contributes to viral pathogenesis through membrane remodeling, immune evasion, and inflammation induction. However, the molecular mechanisms underlying ORF3a‐mediated pathogenesis remain poorly characterized, and no therapeutic strategies targeting ORF3a currently exist. Here, we demonstrate that palmitoylation, a post‐translational modification, governs ORF3a‐mediated viral pathogenesis. Specifically, ORF3a undergoes ZDHHC18‐mediated palmitoylation at evolutionarily conserved Cys130/Cys133 residues, which stabilizes the protein by masking an intrinsic proteasomal degradation signal. This palmitoylation competitively inhibits tripartite motif‐containing 16 (TRIM16)‐dependent K27‐linked polyubiquitination, thereby preventing ORF3a degradation and enhancing viral replication and inflammatory responses. A designed ORF3a‐mimicking palmitoylation‐inhibitory peptide (OPIP) blocked ORF3a palmitoylation, promoted its degradation, and significantly reduced SARS‐CoV‐2 pathogenicity. Collectively, these findings establish ZDHHC18‐mediated palmitoylation as a central regulator of ORF3a stability and virulence, revealing a potentially druggable axis for disrupting SARS‐CoV‐2 pathogenesis.

## Graphical abstract

Palmitoylation by ZDHHC18 blocks ORF3a K27‐linked ubiquitination mediated by TRIM16, thereby preventing its proteasomal degradation and strengthening viral pathogenesis. Targeting palmitoylation through a pharmacological inhibitor (2‐BP), a competitive inhibitory peptide (OPIP), or adenovirus‐mediated knockdown of ZDHHC18 expression presents a potential therapeutic strategy against SARS‐CoV‐2.

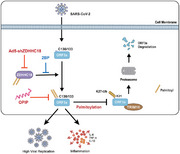

## 1 Introduction

The COVID‐19 pandemic, driven by SARS‐CoV‐2, has been challenges characterized by the virus rapid global transmission, propensity for mutation, significant morbidity and mortality rates, and persistent post‐acute health complications. These factors have spurred extensive studies into the molecular mechanisms behind the pathogenesis of this viral infection. SARS‐CoV‐2 virus possesses a large single‐stranded RNA genome containing multiple open reading frames (ORFs) that encode structural proteins, including the spike (S), envelope (E), membrane (M), and nucleocapsid (N) proteins, as well as eight accessory proteins (ORF 3a, 3b, 6, 7a, 7b, 8, 9b, and 9c) [1]. ORF3a, the largest accessory protein in SARS‐CoV‐2, is exclusive to SARS‐CoV and SARS‐CoV‐2 among the seven known human coronaviruses [2]. This protein was acquired from the β‐CoV lineage and likely originated in bats via viral evolution. Among the intricate ensemble of viral proteins, ORF3a contributes significantly to viral pathogenesis by facilitating viral assembly and release, essential processes in the viral life cycle, while suppressing host antiviral immune responses to enhance viral replication [3, 4, 5, 6]. ORF3a also has been shown to induce excessive inflammation through cellular oxidative stress and the generation of reactive oxygen species, alongside NF‐κB‐mediated TNFα and IL6 production [7, 8, 9, 10]. These processes culminate in apoptotic cell death and tissue damage within the lungs, kidneys, and central nervous system. Furthermore, ORF3a activates the NLRP3 inflammasome, inducing a cytokine storm that significantly contributes to disease severity and mortality [11, 12, 13, 14, 15]. However, the molecular basis of ORF3a‐mediated viral pathogenesis described above remains poorly understood, and no therapeutic strategies targeting ORF3a have been developed to date.

Emerging evidence indicates that coronavirus proteins undergo diverse post‐translational modifications (PTMs) mediated by host enzymes, which significantly influence viral pathogenesis [16]. For instance, palmitoylation and heavy glycosylation of the SARS‐CoV‐2 S protein are critical for viral infectivity and membrane fusion [17, 18]. Acetylation of the SARS‐CoV‐2 N protein disrupts its liquid‐liquid phase separation and impairs its ability to suppress MAVS antiviral signaling [19]. SUMOylation of the N protein regulates viral replication and fitness, while ubiquitination of ORF7a inhibits host IFN‐α signaling by blocking STAT2 phosphorylation [20, 21, 22]. Additionally, glycosylation of ORF8 enables immune evasion by mimicking host MHC‐I molecules [23]. However, the roles and mechanisms of PTMs in modulating ORF3a biological functions remain poorly understood. Palmitoylation is a reversible post‐translational modification involving the attachment of palmitate to cysteine residues via thioester bonds, catalyzed by DHHC palmitoyl acyltransferases to regulate protein trafficking and function [24]. As a key regulator of host‐pathogen interactions, S‐palmitoylation of both viral and host proteins influences pathogen virulence and innate immune responses [25]. Our previous work has demonstrated that palmitoylation stabilizes the E protein oligomer of SARS‐CoV‐2 and enhances its ion channel activity [21]. Given the significant role of the non‐structural protein ORF3a in viral evolution and virus‐host interactions [26, 27], we sought to investigate the modulation of ORF3a by palmitoylation and its resulting functional implications.

In this study, we demonstrate that ORF3a palmitoylation governs ORF3a‐mediated viral pathogenesis. Through cell‐based screening, we identified ZDHHC18 as the primary acyltransferase responsible for catalyzing ORF3a palmitoylation. Furthermore, we show that ZDHHC18‐mediated palmitoylation suppresses TRIM16‐dependent K27 ubiquitination of ORF3a. We also developed an interfering peptide that blocks ORF3a palmitoylation, thereby promoting its proteasomal degradation. Collectively, these findings elucidate the functional and mechanistic roles of SARS‐CoV‐2 ORF3a palmitoylation and propose a novel therapeutic strategy for mitigating SARS‐CoV‐2 infection.

## 2 Results

### 2.1 ORF3a Palmitoylation Determines ORF3a‐Mediated Pathogenesis

**FIGURE 1:**
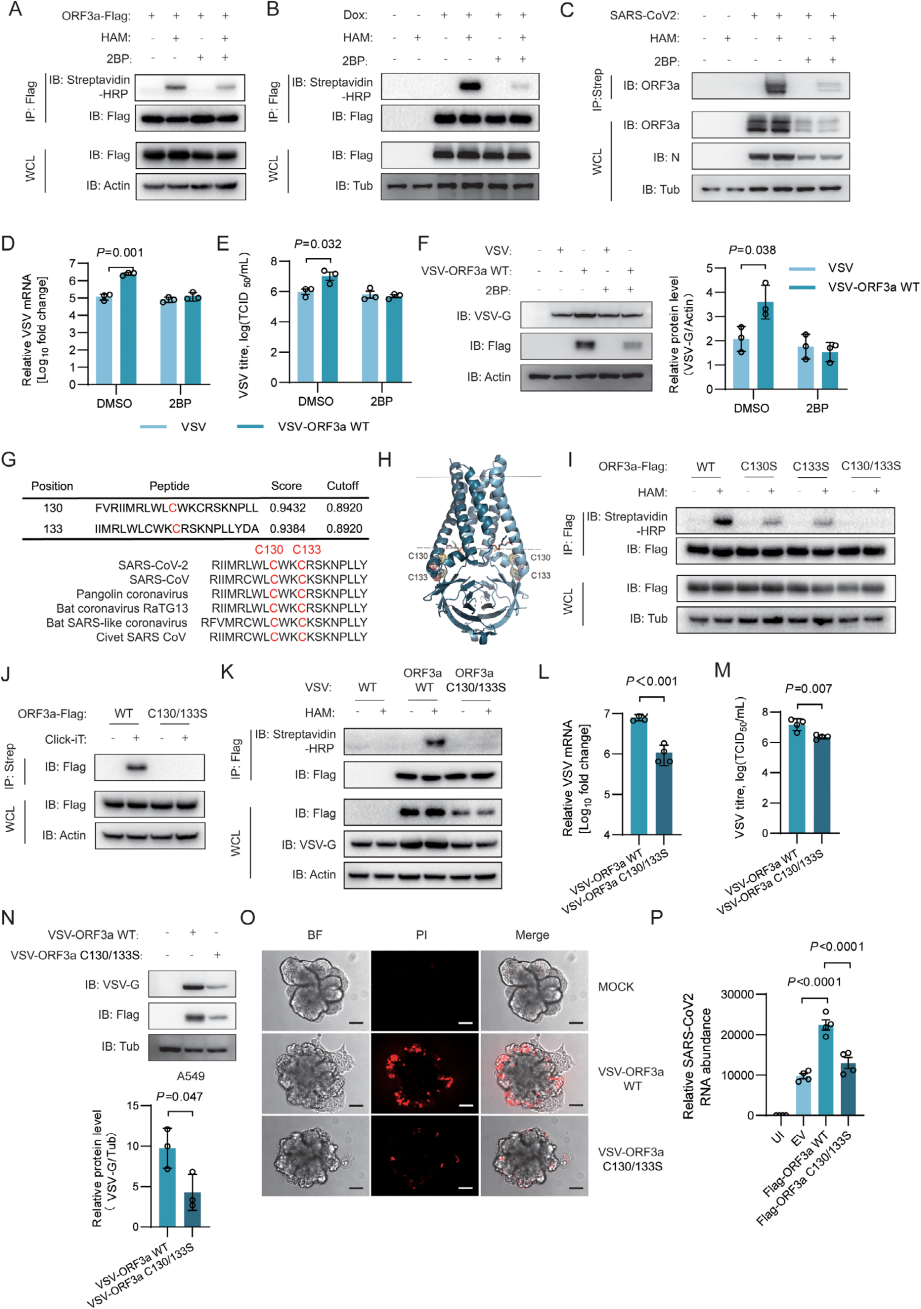
Palmitoylation plays a critical role in ORF3a‐mediated SARS‐CoV‐2 pathogenesis. (A,B) ORF3a palmitoylation levels were assessed byABE assay coupled with immunoblot analysis through transfection (A) or inducible (B) expression of ORF3a. (C) Palmitoylation of endogenous ORF3a in Huh7 cells infected with EG.5 (MOI = 0.01, 48 h) was assessed by ABE assay coupled with immunoblot analysis. (D–F) The fold change in VSV mRNA (D), VSV titers (E) and VSV‐G protein level (F) in A549 cells infected with VSV vs. VSV‐ORF3a WT virus (MOI = 0.1, 16 h). (G) Potential palmitoylation sites were predicted using GPS‐Palm (up), and the sequence conservation of ORF3a among SARS‐like orthologs was analyzed (down). (H) The tertiary structure of ORF3a. (I) ORF3a palmitoylation levels in HEK293T cells transfected with WT or ORF3a mutants were assessed by ABE assay coupled with immunoblot analysis. (J) ORF3a palmitoylation levels in HEK293T cells transfected with WT or ORF3a C130/133S were assessed by the Click‐iT reaction coupled with immunoblot analysis. (K–N) A549 cells were infected with indicated recombinant VSVs (MOI = 0.1, 16 h). ORF3a palmitoylation levels assessed by ABE assay (K). VSV mRNA fold change (L), viral titers (M), and VSV‐G protein levels (N) were compared in A549 cells infected with VSV‐ORF3a WT vs. VSV‐ORF3a C130/133S. (O) Propidium iodide (PI; 1 µg/ml, 10 min) staining in organoids infected with VSV‐ORF3a WT or C130/133S mutant, with scale bars = 50 µm. Representative bright‐field (BF) and immunofluorescence images are shown. (P) The mRNA levels of SARS‐CoV‐2 N gene in 293T‐ACE2 cells transfected with Flag‐ORF3a WT or the C130/133S mutant, followed by SARS‐CoV‐2 EG.5 infection (MOI = 0.01, 48 h). All data are representative of at least three independent experiments. Data are presented as Mean ± SD. Statistical significance was determined by unpaired two‐tailed Student's *t*‐test or one‐way ANOVA. Abbreviations: IP, immunoprecipitation; IB, immunoblot; WCL, whole‐cell lysates; 2BP, 2‐bromopalmitate; HAM, hydroxylamine.

To investigate the palmitoylation of the SARS‐CoV‐2 ORF3a protein, we performed an acyl‐biotin exchange (ABE) assay. As shown in Figure 1A, ectopic expression of ORF3a demonstrated robust palmitoylation. Treatment with2BP, a broad‐spectrum inhibitor of ZDHHC family protein acyltransferases, significantly reduced ORF3a palmitoylation levels. Additionally, we established a stable A549 cell line with doxycycline (Dox)‐inducible ORF3a expression, confirming that ORF3a undergoes palmitoylation in mammalian cells (Figure 1B). Furthermore, we employed another ABE assay to verify endogenous palmitoylation of ORF3a in SARS‐CoV‐2‐infected cells (Figure 1C). Recombinant vesicular stomatitis viruses (VSV) are a well‐established, safe, and versatile viral vector widely employed in the study of viral pathogenesis research due to their capacity to accommodate foreign gene insertions and their robust replication in mammalian cells [28, 29]. Its rapid replication cycle and high‐level protein expression make it particularly suitable for evaluating the impact of individual viral proteins on viral fitness and host responses. There have also been successful precedents in previous studies that employed this model to investigate the function of the SARS‐CoV‐2 N protein [19, 20, 30]. To assess the role of ORF3a palmitoylation in viral pathogenicity, we engineered recombinant VSV‐ORF3a as a surrogate virus (Figure S1A). Infection of A549 cells with these recombinant VSVs at a multiplicity of infection (MOI) of 0.1 for 12 or 24 h revealed that VSV‐ORF3a significantly enhanced viral replication, as indicated by increased VSV‐specific mRNA levels, viral titers, and VSV‐G protein expression compared to the control virus (Figure S1B–D). These findings were corroborated in an organoid‐based infection model (Figure S1E–G), consistent with prior reports that ORF3a deletion reduces viral load [31]. Given the robust palmitoylation of ORF3a, we examined its impact on recombinant VSV infection. Treatment with 2BP significantly decreased VSV‐ORF3a‐specific mRNA levels, viral titers, and VSV‐G protein expression to levels comparable with the control virus (Figure 1D–F), indicating that ORF3a palmitoylation contributed significantly to the pathogenesis of the surrogate virus and might also be critical for SARS‐CoV‐2 pathogenesis.

To identify the palmitoylation sites of ORF3a, we used the motif‐based prediction tool GPS‐Palm, which identified two candidate cysteine residues at positions 130 and 133. Sequence alignment revealed these residues are conserved within palmitoylation motifs across SARS‐like orthologs and all the SARS‐CoV‐2 variants (Figure 1G; Figure S1H). Structural analysis showed that Cys130 and Cys133 are positioned within a cysteine‐rich pocket near the transmembrane domain (Figure 1H). We generated point mutants of ORF3a, and the simultaneous substitution of Cys130 and Cys133 with serine (C130/133S) markedly reduced palmitoylation, as confirmed by ABE assays and Click‐iT labeling (Figure 1I,J). These residues appear to serve as primary palmitoylation sites that may compensate for each other when singly mutated. The C130/133S mutant exhibited significantly reduced palmitoylation compared to VSV‐ORF3a WT (Figure 1K), which correlated with impaired viral replication and decreased secretion of inflammatory and interferon‐related cytokines (TNFα, IFNβ, IL1β, and IL6) (Figure 1L–N; Figure S1I). Similar results were obtained in organoid models (Figure S2). Visualization of infected organoids revealed more severe structural collapse and cell death induced by VSV‐ORF3a WT vs. the C130/133S mutant (Figure 1O). In SARS‐CoV‐2–infected cells, overexpression of ORF3a increased viral replication, whereas cells overexpressing the C130/133S mutant showed no significant change compared to controls (Figure 1P). Collectively, these findings establish that Cys130 and Cys133 are the primary palmitoylation sites of ORF3a. The C130/133S mutation phenocopies 2BP treatment, indicating that ORF3a palmitoylation is critical for mediating its pathogenic effects both in surrogate virus and SARS‐CoV‐2 infection.

### 2.2 ZDHHC18 is the Main Palmitoyl Transferases for ORF3a Palmitoylation

**FIGURE 2:**
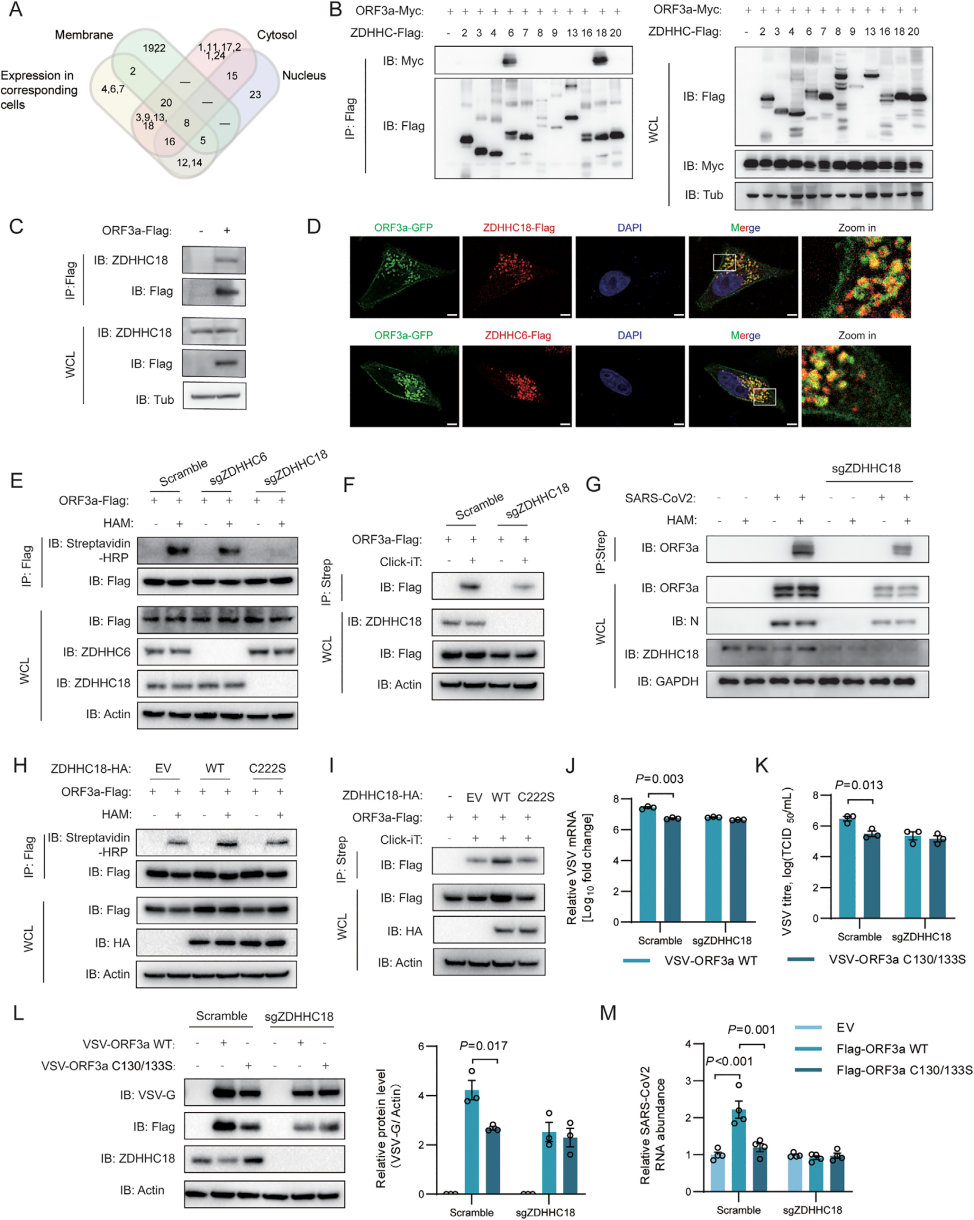
ORF3a palmitoylation is mediated by ZDHHC18. (A) Venn diagram showing the expression patterns of different ZDHHCs in A549 and HEK293T cells, including expression levels and subcellular distributions. (B) Immunoblot analysis of WCL and anti‐Flag immunoprecipitant from HEK293T cells transfected with ZDHHCs‐Flag and ORF3a‐Myc. (C) Interactions between ORF3a and endogenous ZDHHC18 were assessed by co‐immunoprecipitation in HEK293T cells. (D) Colocalization of ZDHHC18‐Flag or ZDHHC6‐Flag and ORF3a‐GFP in transfected HeLa cells was assessed by immunofluorescence (DAPI counterstain; scale bar = 20 µm). (E) ORF3a palmitoylation levels in WT, sgZDHHC6 and sgZDHHC18 HEK293T cells were assessed by ABE assay coupled with immunoblot analysis. (F) ORF3a palmitoylation levels in scramble control vs. sgZDHHC18 HEK293T cells transfected with ORF3a‐Flag were assessed by the Click‐iT reaction coupled with immunoblot analysis. (G) Endogenous ORF3a palmitoylation levels in scramble control or sgZDHHC18 Huh7 cells infected with EG.5 strain (MOI = 0.01, 48 h) were assessed by ABE assay coupled with immunoblot analysis. (H,I) ORF3a palmitoylation levels in HEK293T cells transfected with indicated plasmids were assessed by ABE assay (H) and Click‐iT reaction (I) coupled with immunoblot analysis. (J–L) The fold changes in VSV mRNA (J), VSV titers (K) and VSV‐G protein level (L) in scramble control vs. sgZDHHC18 A549 cells infected with VSV‐ORF3a WT or VSV‐ORF3a C130/133S (MOI = 0.1, 16 h). (M) The mRNA levels of SARS‐CoV‐2 N gene in scramble control vs. sgZDHHC18 293T‐ACE2 cells transfected with ORF3a‐Flag WT or the C130/133S mutant, followed by SARS‐CoV‐2 EG.5 infection (MOI = 0.01, 48 h). All data are representative of at least three independent experiments with similar results. Data are presented as Mean ± SD. Statistical significance was determined by unpaired two‐tailed Student's *t*‐test or one‐way ANOVA. Abbreviations: IP, immunoprecipitation; WCL, whole‐cell lysates; HAM, hydroxylamine.

To identify the predominant palmitoyl transferases for ORF3a in cells, we implemented a multi‐step screening strategy integrating protein expression analysis, subcellular localization assessment, and co‐immunoprecipitation. First, using data from the Human Protein Atlas, we assessed the expression of all ZDHHC family members in A549 and 293T cell lines (Figure S3A,B). Exclusion of nuclear‐localized ZDHHCs, which lacked spatial overlap with ORF3a, identified candidate genes for experimental validation: ZDHHC2, 3, 4, 6, 7, 8, 9, 13, 16, 18, and 20 (Figure 2A). We then co‐transfected 293T cells with ORF3a‐Myc and ZDHHCs‐Flag plasmids to screen for interacting partners. Results revealed specific binding between ORF3a and ZDHHC6, or ZDHHC18 (Figure 2B). Endogenous co‐immunoprecipitation and confocal microscopy confirmed robust interactions between ORF3a and both ZDHHC6 and ZDHHC18 (Figure 2C,D; Figure S3C). However, deficiency of ZDHHC18 significantly reduced ORF3a palmitoylation levels relative to ZDHHC6 knockdown (Figure 2E). Using click chemistry, we further demonstrated that ZDHHC18 deficiency substantially decreased ORF3a palmitoylation (Figure 2F). The level of palmitoylation modification of ORF3a after SARS‐CoV‐2 infection also decreases with the knockdown of ZDHHC18 (Figure 2G). Subsequent experiments showed that ectopic expression of wild‐type (WT) ZDHHC18, but not its catalytically inactive C222S mutant, enhanced ORF3a palmitoylation, as validated by ABE assay and Click‐iT labeling (Figure 2H,I). In contrast, ectopic ZDHHC6 expression did not affect ORF3a palmitoylation (Figure S3D). Notably, while a marginal increase in ZDHHC18 expression occurred in lung tissues of SARS‐CoV‐2‐infected K18‐hACE2 mice, cellular transfection with ORF3a revealed no significant alterations in ZDHHC18 expression at mRNA or protein levels (Figure S3E–G). These results indicate that the catalytic activity of ZDHHC18, rather than its protein expression level, is required for ORF3a palmitoylation. Furthermore, ZDHHC18 deficiency abolished the replication differences between VSV‐ORF3a WT and VSV‐ORF3a C130/133S (Figure 2J–L), an effect not observed with ZDHHC6 deficiency (Figure S3H–J). In SARS‐CoV‐2‐infected cells, the enhanced viral replication resulting from ORF3a overexpression was suppressed by ZDHHC18 knockdown (Figure 2M). Similarly, ZDHHC18 deficiency markedly reduced inflammatory and IFN‐associated cytokine levels (TNFα, IFNβ, IL1β, and IL6) induced by VSV‐ORF3a WT, rendering them comparable to the VSV‐ORF3a C130/133S group (Figure S3K). Collectively, these findings establish ZDHHC18 as the major acyltransferase mediating ORF3a palmitoylation.

### 2.3 ZDHHC18‐Mediated Palmitoylation Protects the ORF3a From Proteasomal Degradation

**FIGURE 3:**
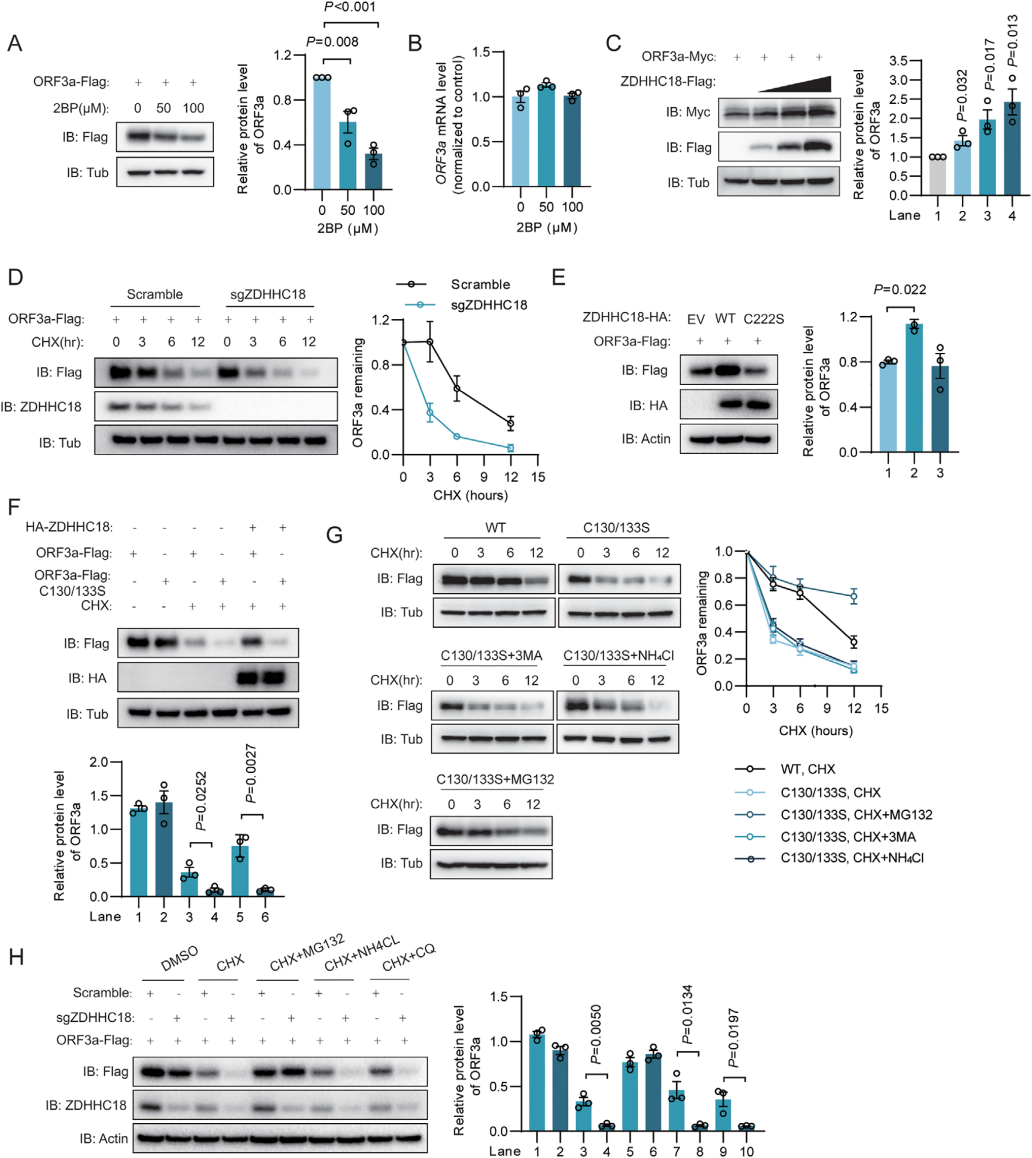
Palmitoylation inhibits ORF3a degradation via the proteasome pathway. (A,B) ORF3a expression in cells treated with the 2BP was evaluated at the protein level by immunoblot (A) and at the transcriptional level by qPCR (B). (C) ORF3a expression in HEK293T cells transfected with ORF3a‐Myc and increasing doses of ZDHHC18‐Flag were assessed by immunoblot analysis. (D) Immunoblot analysis of ORF3a expression in scramble control vs. sgZDHHC18 HEK293T cells transfected with ORF3a‐Flag and then treated with CHX for the indicated durations. (E) ORF3a expression in HEK293T cells co‐transfected with ORF3a‐Flag and either WT ZDHHC18‐HA or the enzymatically inactive ZDHHC18‐HA C222S mutant were assessed by immunoblot analysis. (F) The expression of ORF3a or its C130/133S mutant in HEK293T cells co‐transfected with ZDHHC18‐HA and CHX treatment was assessed by immunoblot analysis. (G) ORF3a expression in HEK293T cells transfected with the indicated plasmids was assessed by immunoblotting after treatment with CHX alone or in combination with MG132, 3‐MA, or NH4Cl for the indicated durations. (H) ORF3a expression in scramble control vs. sgZDHHC18 HEK293T cells transfected with ORF3a‐Flag was assessed by immunoblotting after treatment with CHX alone or in combination with MG132, CQ, or NH4Cl. All data are representative of at least three independent experiments with similar results. Data are presented as Mean ± SD. Statistical significance was determined by unpaired two‐tailed Student's *t*‐test or one‐way ANOVA. Abbreviations: IB, immunoblot; 3‐MA, 3‐methyladenine; CQ, chloroquine.

We further assessed ORF3a stability mediated by ZDHHC18‐mediated palmitoylation. Inhibition of palmitoylation with 2BP reduced ORF3a protein levels in dose‐(Figure 3A) and time‐dependent manner (Figure S4A), while ORF3a mRNA levels remained unchanged (Figure 3B; Figure S4B). ZDHHC18 overexpression significantly increased ORF3a protein abundance (Figure 3C). Cycloheximide (CHX) chase assays demonstrated accelerated ORF3a degradation upon ZDHHC18 deficiency (Figure 3D), while ZDHHC18 overexpression substantially stabilized ORF3a (Figure S4C). Notably, the catalytically inactive ZDHHC18‐C222S mutant exhibited diminished ORF3a stabilization compared to wild‐type (Figure 3E). The C130/133S mutation accelerated ORF3a degradation (Figure S4D), mirroring the degradation kinetics observed under ZDHHC18 deficiency. Consequently, ZDHHC18 overexpression failed to rescue the instability of the ORF3a C130/133S mutant (Figure 3F), indicating that ZDHHC18‐mediated palmitoylation restricts ORF3a degradation.

The accelerated degradation of palmitoylation‐deficient ORF3a C130/133S was rescued by proteasomal inhibitor MG132, but not by lysosomal inhibitors (NH4Cl) or autophagy inhibitor 3‐methyladenine (3‐MA) (Figure 3G). Similarly, in ZDHHC18‐knockdown 293T cells expressing ORF3a, destabilization was reversed by MG132 but not by NH4Cl or chloroquine (CQ) (Figure 3H). Conversely, while ZDHHC18 overexpression inhibited ORF3a degradation, this stabilization was counteracted by proteasomal inhibitors MG132 or bortezomib (BTZ) (Figure S4E,F). Collectively, these results demonstrate that ZDHHC18‐mediated palmitoylation stabilizes ORF3a by antagonizing proteasomal degradation.

### 2.4 ZDHHC18‐Mediated Palmitoylation Restricts TRIM16‐Mediated K27 Ubiquitination of ORF3a

**FIGURE 4:**
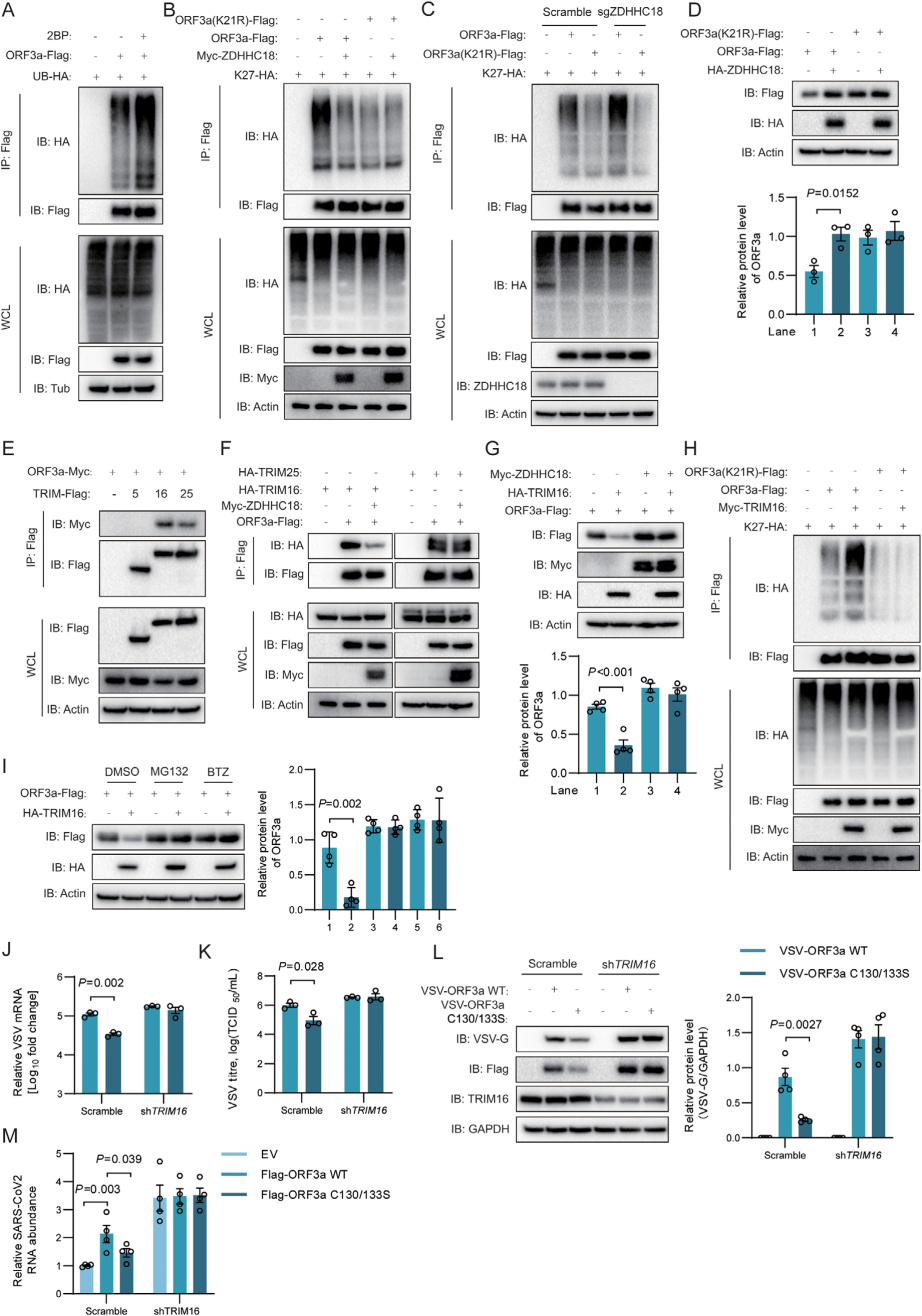
Palmitoylation of ORF3a prevents its K27‐linked ubiquitination mediated by TRIM16. (A) Immunoblot analysis of ORF3a ubiquitination in HEK293T cells transfected with ORF3a and HA‐Ub plasmid as indicated after the treatment of 2BP. Cells were treated with MG‐132 for 4 h before sample collection. (B) Immunoblot analysis of K27‐linked ubiquitination of ORF3a in HEK293T cells transfected with Myc‐ZDHHC18 and the indicated ORF3a‐Flag constructs. Cells were treated with MG‐132 for 4 h before sample collection. (C) Immunoblot analysis of K27‐linked ubiquitination of ORF3a in scramble control and sgZDHHC18 HEK293T cells transfected with ORF3a‐Flag or the ORF3a‐Flag K21R. Cells were treated with MG‐132 for 4 h before sample collection. (D) Immunoblot analysis of ORF3a expression in HEK293T cells co‐transfected with HA‐ZDHHC18 and the indicated ORF3a‐Flag constructs. (E) Immunoblot analysis of WCL and anti‐Flag immunoprecipitant from HEK293T cells transfected with ORF3a‐Myc and TRIM5, TRIM16 or TRIM25‐Flag. (F) Immunoblot analysis of WCL and anti‐Flag immunoprecipitant from HEK293T cells transfected with ORF3a‐Flag, Myc‐ZDHHC18, and HA‐TRIM16 or HA‐TRIM25. (G) Immunoblot analysis of ORF3a expression in HEK293T cells transfected with ORF3a‐Flag, Myc‐ZDHHC18 and HA‐TRIM16. (H) Immunoblot analysis of K27‐linked ubiquitination of ORF3a in HEK293T cells transfected with Myc‐TRIM16 and ORF3a‐Flag or ORF3a‐Flag K21R. Cells were treated with MG‐132 for 4 h before sample collection. (I) Immunoblot analysis of ORF3a expression in HEK293T cells transfected with ORF3a‐Flag and HA‐TRIM16 and then treated with proteasome inhibitors MG132 or BTZ. (J–L) The fold change of VSV mRNA (J), VSV titers (K) and VSV‐G protein level (L) in scramble control or shRNAs for TRIM16 in A549 cells infected with VSV‐ORF3a WT or VSV‐ORF3a C130/133S (MOI = 0.1, 16 h). (M) The mRNA levels of SARS‐CoV‐2 N gene in scramble control vs. shTRIM16 293T‐ACE2 cells transfected with ORF3a WT or the ORF3a C130/133S, followed by SARS‐CoV‐2 EG.5 infection (MOI = 0.01, 48 h). All data are representative of at least three independent experiments with similar results. Data are presented as Mean ± SD. Statistical significance was determined by unpaired two‐tailed Student's *t*‐test or one‐way ANOVA. Abbreviations: IP, immunoprecipitation; IB, immunoblot; WCL, whole‐cell lysates; BTZ, bortezomib.

To investigate how palmitoylation regulates ORF3a proteasomal degradation, we assessed its impact on ORF3a ubiquitination. 2BP‐induced palmitoylation blockade markedly increased ORF3a ubiquitination, while ZDHHC18 overexpression significantly decreased it (Figure 4A; Figure S5A). Immunoprecipitation of ORF3a from 293T cells coupled with mass spectrometry identified K21 as the critical ubiquitination site (Figure S5B). Cycloheximide chase assays revealed that the ORF3a‐K21R mutant exhibited substantially extended half‐life compared to wild‐type controls. Consistent with this finding, ORF3a‐K21R displayed slower degradation kinetics during 2BP treatment (Figure S5C). Characterization of ubiquitin linkage types using linkage‐specific mutants showed that the K21R mutation selectively abrogated K27‐linked polyubiquitination without affecting other linkage types (K6, K11, K29, K33, K48, or K63; Figure S5D). The palmitoylation‐deficient ORF3a C130/133S mutant exhibited enhanced K27‐linked polyubiquitination (Figure S5E). Immunoprecipitation assays demonstrated that ZDHHC18 suppressed K27‐linked polyubiquitination of wild‐type ORF3a but not the K21R or C130/133S mutants (Figure 4B,C; Figure S5F). Correspondingly, ZDHHC18 enhanced wild‐type ORF3a expression but not ORF3a‐K21R (Figure 4D), indicating that ZDHHC18‐mediated palmitoylation inhibits K27‐linked polyubiquitination of ORF3a.

Previous studies have indicated that ORF3a interacts with E3 ubiquitin ligases TRIM5, TRIM16, and TRIM25 (COVID‐19 Interactome Database) [32]. We hypothesized that ZDHHC18‐mediated palmitoylation competitively regulates ORF3a ubiquitination. Co‐immunoprecipitation assays revealed ORF3a interaction with TRIM16 and TRIM25 (Figure 4E). Crucially, ZDHHC18 overexpression attenuated TRIM16 binding to ORF3a without affecting TRIM25 binding (Figure 4F). Interaction between TRIM16 and ORF3a C130/133S was diminished compared to wild‐type ORF3a (Figure S5G). Increasing TRIM16 expression progressively reduced ORF3a levels (Figure S5H), an effect rescued by ZDHHC18 overexpression (Figure 4G). TRIM16 overexpression enhanced K27‐linked ubiquitination of wild‐type ORF3a but not the K21R mutant (Figure 4H), and TRIM16‐induced degradation was blocked by proteasome inhibitors (MG132 or BTZ) (Figure 4I). Finally, TRIM16 deficiency abrogated replication differences between VSV‐ORF3a WT and VSV‐ORF3a C130/133S (Figure 4J–L). TRIM16 deficiency eliminated the replication differences between SARS‐CoV‐2–infected cells overexpressing ORF3a and those overexpressing the C130/133S mutant (Figure 4M). Collectively, these findings establish that ZDHHC18 competitively inhibits TRIM16‐mediated K27‐linked polyubiquitination to regulate ORF3a stability during viral infection.

### 2.5 ZDHHC18‐Mediated ORF3a Palmitoylation is Critical for the Viral Pathogenesis

**FIGURE 5:**
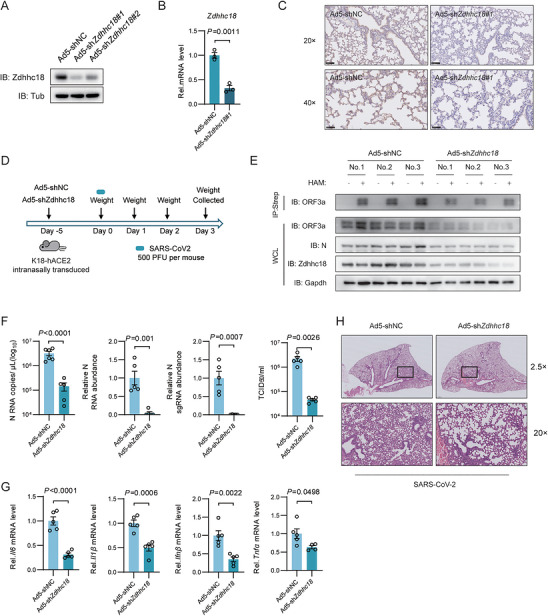
ZDHHC18‐mediated ORF3a palmitoylation exacerbated viral pathogenesis in vivo. (A,B) Hepa1‐6 cells were infected with Ad5‐sh*Zdhhc18* for 48 h, and knockdown efficiency was confirmed by (A) IB analysis of ZDHHC18 protein levels and (B) qPCR analysis of *Zdhhc18* mRNA levels. (C) Immunohistochemistry assay of ZDHHC18 protein level of lung sections from Balb/c mice infected with 2 × 10^8^ FFU Ad5‐sh*Zdhhc18* for 5 days. Scale bar, 100 µm (top) and 50 µm (bottom). (D) The schematic diagrams of Ad5‐shZdhhc18 transduced mice and subsequent SARS‐CoV2 challenge. (E–H) Ad5‐Zdhhc18 transduced K18‐hACE2 mice were intranasally infected with 500 PFU of SARS‐CoV2 (Wuhan‐Hu1) in 50 µL DMEM. (E) Endogenous ORF3a palmitoylation levels in lung tissues were assessed by ABE assay coupled with immunoblot analysis. (F) SARS‐CoV‐2 genome copy numbers (targeting the N gene), N sgRNA levels and SARS‐CoV‐2 titers in lungs were measured at 3 dpi. (G) Cytokine Il6, Il1β, Ifnβ, and Tnfα mRNA levels in lung homogenates at 3 dpi were measured by qPCR. (H) The tissue injury in lung sections was analyzed by hematoxylin and eosin staining. Scale bar, 1 mm (Top) or 100 µm (Down). All data are representative of at least three independent experiments with similar results. Data are presented as Mean ± SD. Statistical significance was determined by unpaired two‐tailed Student's t‐test. Abbreviations: IB, immunoblot.

To investigate the biological function of ZDHHC18‐mediated ORF3a palmitoylation in vivo, we generated an adenoviral shRNA vector (Ad5‐sh*Zdhhc18*) targeting Zdhhc18 expression [33, 34]. Knockdown efficiency was confirmed in vitro by immunoblotting and qPCR, demonstrating Ad5‐sh*Zdhhc18*#1 significantly reduced Zdhhc18 protein and mRNA levels in Ad5‐sh*Zdhhc18*‐transduced cells compared to Ad5‐shNC controls (Figure 5A,B). This efficiency was validated in vivo (Figure 5C), with Ad5‐sh*Zdhhc18*‐transduced animals exhibiting significantly suppressed Zdhhc18 expression relative to Ad5‐shNC controls, demonstrating effective knockdown both in vitro and in vivo.

Consequently, 6–8‐weeks‐old Balb/c mice were intranasally transduced with 2 × 10^8^ FFU of Ad5‐sh*Zdhhc18* or Ad5‐shNC. 5 days post‐transduction, mice were infected with recombinant VSVs (Figure S6A). While no significant differences in body weight changes were observed among groups (Figure S6B), lung VSV‐specific mRNA levels were significantly higher in VSV‐ORF3a WT‐infected mice compared to VSV‐ORF3a C130/133S‐infected controls. Crucially, Zdhhc18 knockdown abolished these differences (Figure S6C,D). Correspondingly, levels of Tnfα, Ifnβ, Il1β, and Il6 mRNA in lungs of VSV‐ORF3a C130/133S‐infected mice were significantly lower than in VSV‐ORF3a WT‐infected mice. Zdhhc18 knockdown markedly reduced cytokine levels induced by VSV‐ORF3a WT to levels comparable with the VSV‐ORF3a C130/133S group (Figure S6E–H). Furthermore, H&E staining revealed greater immune cell infiltration and more severe tissue injury in the lungs of VSV‐ORF3a WT‐infected mice vs. VSV‐ORF3a C130/133S‐infected controls. Lung‐specific Zdhhc18 knockdown induced comparable inflammatory infiltration in response to both recombinant VSVs (Figure S6I). Similarly, we achieved specific knockdown of *Zdhhc18* in the lung tissues of K18‐hACE2 mice via intranasal instillation of Ad5‐sh*Zdhhc18* or the control Ad5‐shNC virus, followed by infection with SARS‐CoV‐2 (Wuhan‐Hu1) virus to directly assess the role of the host palmitoyl transferase ZDHHC18 in SARS‐CoV‐2 pathogenesis (Figure 5D). We first assessed ORF3a palmitoylation by comparing its signal intensity in the Ad5‐shZdhhc18 group relative to the control group. This result shows that knockdown of Zdhhc18 markedly reduced ORF3a palmitoylation, corresponding to lower viral N protein levels in vivo (Figure 5E). Consistent with our findings in the VSV‐ORF3a chimeric virus model, the results show that lung‐specific knockdown of *Zdhhc18* in K18‐hACE2 mice significantly reduced pulmonary viral load, mitigated the infection‐triggered elevation of cytokines, and alleviated SARS‐CoV‐2 infection‐induced lung histopathological damage (Figure 5F–H). Collectively, these findings demonstrate that ZDHHC18‐mediated ORF3a palmitoylation is critical for viral pathogenesis in vivo.

### 2.6 Targeting ORF3a Palmitoylation Restricts Pathogenesis During SARS‐CoV‐2 Infection

**FIGURE 6:**
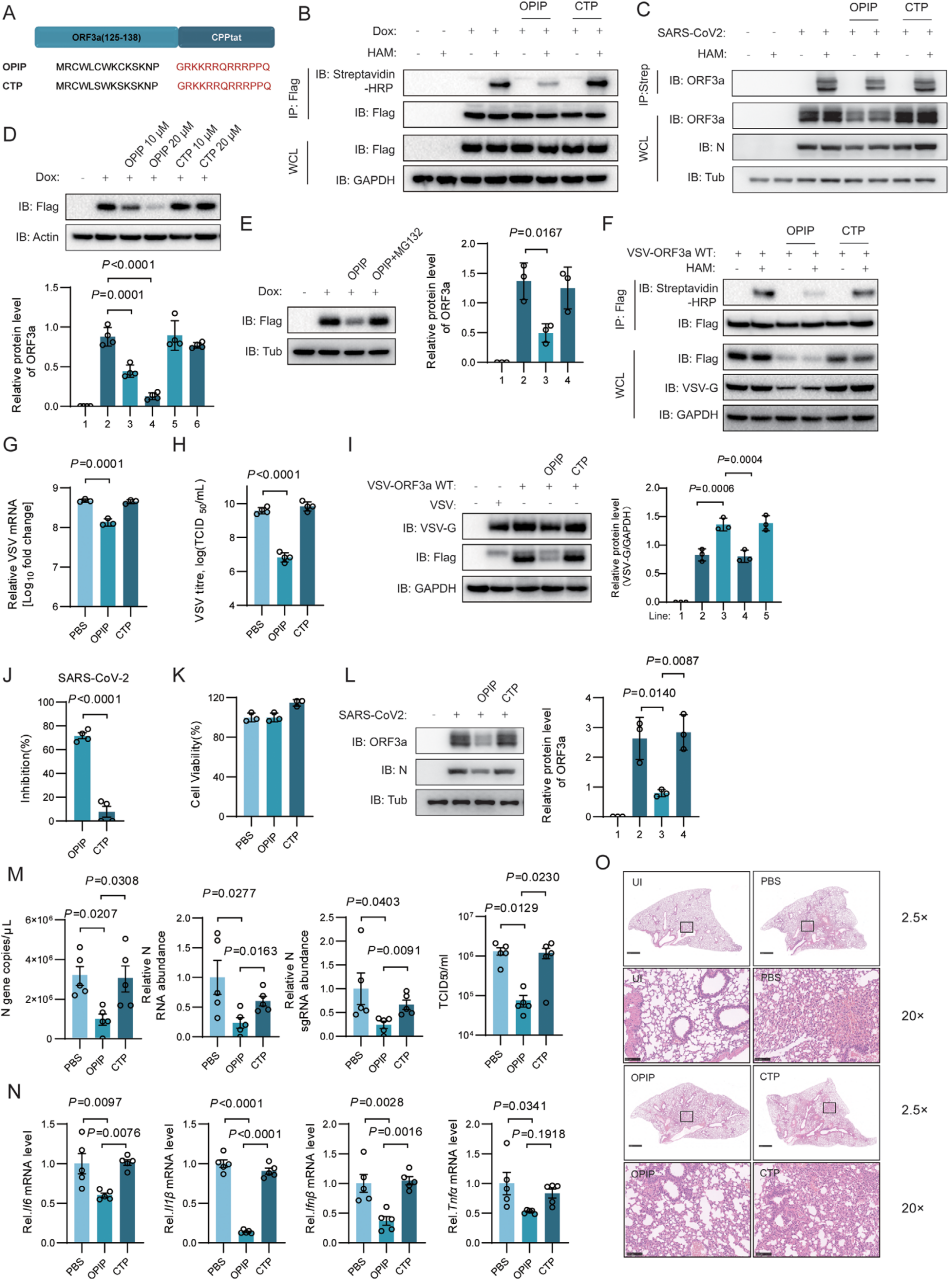
The peptide OPIP targeting ORF3a palmitoylation limits SARS‐CoV‐2 infection. (A) Amino acid sequences of interfering peptides targeting C130/133 residue of ORF3a with cell penetrating peptide (OPIP) and CTP as a negative control. (B) ORF3a palmitoylation levels in inducible ORF3a expression A549 cells were assessed by ABE assay coupled with immunoblot analysis after treatment with 20 µm OPIP or CTP. (C) Endogenous ORF3a palmitoylation levels in Huh7 cells infected with the EG.5 strain (MOI = 0.01, 48 h) were assessed by ABE assay coupled with immunoblot analysis. (D) ORF3a expression in inducible A549 cells was assessed by immunoblotting after treatment with increasing concentrations of OPIP or CTP. (E) ORF3a expression in inducible A549 cells was assessed by immunoblotting after treatment with 20 µM OPIP in the presence or absence of MG132. (F) ORF3a palmitoylation levels in A549 cells infected with VSV‐ORF3a WT (MOI = 0.1, 16 h) were assessed by ABE assay coupled with immunoblot analysis after treatment with 20 µm OPIP or CTP. (G–I) The fold change of VSV mRNA (G), VSV titers (H) and VSV‐G protein level (I) in A549 cells infected with VSV‐ORF3a WT (MOI = 0.1, 16 h) and incubated with 20 µm OPIP or CTP. (J) Vero‐E6 cells were infected with EG.5 strain (MOI = 0.01, 48 h) and treated with 20 µm OPIP or CTP. Viral inhibition rate was calculated through SARS‐CoV‐2 genome copy numbers (primer target the N gene). (K) Cytotoxicity of 20 µm OPIP or CTP in Vero‐E6 cells was detected based on the CCK8 assay. (L) IB analysis of ORF3a and N protein expression in Huh7 cells infected with the EG.5 strain (MOI = 0.01, 48 h) after treatment with 20 µM OPIP or CTP. (M–O) K18‐hACE2 mice were intranasally inoculated with SARS‐CoV‐2 (EG.5, 10^6^ PFU per mouse) and treated with PBS, OPIP, or CTP (20 mg/kg, i.p., QD). (M) SARS‐CoV‐2 genome copy numbers (targeting the N gene), N sgRNA levels, and SARS‐CoV‐2 titers in lungs were measured at 3 dpi. (N) Cytokine *Il6*, *Il1β*, *Ifnβ*, and *Tnfα* mRNA levels in lung homogenates at 3 dpi were measured by qPCR. (O) The tissue injury in lung sections was analyzed by hematoxylin and eosin staining. Scale bar, 1 mm (top) or 100 µm (bottom). All data are representative of at least three independent experiments with similar results. Data are presented as Mean ± SD. Statistical significance was determined by unpaired two‐tailed Student's *t*‐test or one‐way ANOVA. Abbreviations: IP, immunoprecipitation; IB, immunoblot; ABE, acyl‐biotin exchange; Dox, doxycycline.

Competitive inhibition represents a well‐established strategy for targeting specific enzymatic activities. Based on the identified palmitoylation motif in ORF3a, we designed an ORF3a‐mimicking palmitoylation‐inhibitory peptide (OPIP) to competitively inhibit ORF3a palmitoylation. Inhibitors were generated by fusing a cell‐penetrating peptide to the wild‐type ORF3a motif sequence (residues 125–138) or to a control sequence harboring C130/133S mutations (CTP) (Figure 6A). ABE assays demonstrated that OPIP significantly reduced ORF3a palmitoylation (Figure 6B,C). In A549 cells with Dox‐inducible ORF3a expression, OPIP treatment, but not CTP, dose‐dependently decreased ORF3a protein levels, an effect reversible by the proteasomal inhibitor MG132 (Figure 6D,E). These results establish OPIP as a competitive inhibitor of ORF3a palmitoylation, which promotes ubiquitin‐mediated degradation of ORF3a.

To assess OPIP's antiviral efficacy, cells were pretreated prior to VSV‐ORF3a WT infection. OPIP significantly reduced palmitoylation signals, VSV‐specific mRNA levels, VSV‐G protein expression, and viral titers (Figure 6F–I) while also inhibiting authentic SARS‐CoV‐2 (strain EG.5) replication without affecting cell viability (Figure 6J,K). Similarly, In SARS‐CoV‐2‐infected cells, OPIP treatment, but not CTP, decreased endogenous ORF3a protein levels (Figure 6L). In vivo, K18‐hACE2 mice pretreated intraperitoneally with OPIP, CTP, or PBS were infected with SARS‐CoV‐2 (EG.5 or Wuhan‐Hu1). At 3 dpi, OPIP treatment significantly inhibited ORF3a palmitoylation in lung tissues (Figure S7A). OPIP‐treated mice exhibited significantly reduced lung viral genomic RNA levels and viral titers (approximately one‐log reduction) compared to PBS‐ or CTP‐treated groups (Figure 6M; Figure S7B), accompanied by attenuated expression of Tnfα, Ifnβ, Il1β, and Il6 (Figure 6N; Figure S7C). Histological analysis confirmed that OPIP mitigated SARS‐CoV‐2‐induced lung pathology—including alveolar septal thickening and inflammatory‐cell infiltration—observed in control groups (Figure 6O; Figure S7D). Collectively, these findings demonstrate that OPIP disrupts ORF3a palmitoylation and significantly mitigates SARS‐CoV‐2 pathogenicity in vitro and in vivo.

## 3 Discussion

Our study provides compelling evidence that the SARS‐CoV‐2 accessory protein ORF3a is palmitoylated at Cys130 and Cys133, a modification essential for its pro‐pathogenic functions. This conclusion is supported by multiple lines of evidence, including ABE assays, Click‐iT labeling, mutational analysis, pharmacological inhibition, and in vivo inhibition assays with a novel substrate‐mimetic peptide. Disruption of ORF3a palmitoylation consistently attenuated viral replication, reduced inflammatory cytokine production, and mitigated tissue damage across diverse experimental models (Figure S7E). These results add to a growing body of work indicating that host‐mediated post‐translational modifications of coronavirus proteins (for example, S‐protein palmitoylation and N‐protein acetylation/SUMOylation) meaningfully influence infectivity and immune evasion [17, 19, 20, 35]. This reinforces the premise that host‐mediated PTMs are central modulators of coronavirus biology which parallels to other viral systems, including influenza hemagglutinin, HIV‐1 gp41, and flavivirus structural proteins, where palmitoylation regulates membrane fusion, budding, and assembly, reinforcing an evolutionarily conserved role for lipidation in viral protein fate and pathogenic potential [25, 36, 37]. Thus, our data not only establish palmitoylation as decisive for ORF3a function but also situate ORF3a within a broader, evolutionarily conserved paradigm in which lipidation governs viral protein fate and pathogenic potential.

Mechanistically, we identified ZDHHC18 as the primary acyltransferase responsible for ORF3a palmitoylation. This modification at Cys130/133 appears to sterically hinder TRIM16‐mediated K27‐linked polyubiquitination at Lys21, thereby preventing proteasomal degradation of ORF3a. The specificity of this regulatory mechanism is underscored by several findings: ZDHHC18 loss or catalytic inactivation accelerates ORF3a degradation and attenuates pathogenic readouts; catalytically inactive ZDHHC18 cannot restore ORF3a stability; K21R substitution abolishes detectable K27‐linked ubiquitination; and TRIM16 is required for ORF3a degradation. These observations align with established models in ubiquitin biology, where specific ubiquitin linkage types (e.g., K27, K48, and K63) encode distinct functional outcomes. Notably, while K27‐linked ubiquitination has been implicated in non‐proteolytic signaling and selective autophagy in most cases, its role in ORF3a turnover suggests nuanced crosstalk between ubiquitin signaling and palmitoylation‐mediated protection from degradation [38]. In our study, the involvement of K27‐linked chains is inferred from linkage‐specific mutant constructs and the selective loss of K27‐linked signal upon K21R substitution, and thus primarily reflects the behavior of K27 linkages under these experimental conditions rather than a comprehensive exclusion of all other lysine residues or linkage types. Based on mass spectrometry results, we consistently identified Lys21 as an important ubiquitination site of ORF3a, therefore, K21 represents a key residue for TRIM16‐dependent regulation of ORF3a. However, as we have not performed exhaustive quantitative analysis of all potential lysine modifications, we cannot exclude the possibility of low‐level or context‐dependent ubiquitination at other sites. Together, these data define palmitoylation as a protective PTM that functionally antagonizes a host ubiquitin ligase pathway to preserve ORF3a levels, thereby augmenting viral fitness and inflammatory potential.

These mechanistic insights have implications for SARS‐CoV‐2 pathogenesis. We propose that the stabilization of palmitoylated ORF3a promotes viral replication and amplifies host inflammatory responses, potentially contributing to tissue damage, inflammasome activation, and cytokine storm observed in severe COVID‐19. Clinical plausibility for this model is provided by case reports from severe COVID‐19 patients showing elevated ORF3a expression and heightened inflammatory signatures [39, 40]. Furthermore, parallels with other viral accessory proteins that modulate host ubiquitin pathways, such as HIV‐1 Vpu's manipulation of β‐TrCP to downregulate Tetherin [41], underscore a recurring viral strategy of co‐opting or evading host ubiquitin systems. Importantly, ZDHHC18 knockdown or OPIP‐mediated competitive inhibition uniformly attenuated these pathological outcomes in both VSV‐ORF3a and authentic SARS‐CoV‐2 infection models, demonstrating that interrupting the palmitoylation–stability axis suppresses ORF3a driven pathogenicity in biologically relevant contexts. Our results highlight several actionable strategies. Targeting host ZDHHC18 could lower ORF3a abundance with a potentially high barrier to viral resistance. This strategy mirrors successful host‐directed antiviral approaches in other infections, such as CCR5 antagonists for HIV and host kinase inhibitors for HCV [42, 43]. Substrate‐mimetic peptides (OPIP) effectively compete for palmitoylation, promote ORF3a degradation, and decreased lung viral load and inflammation in K18‐hACE2 mice, providing proof‐of‐concept that targeted inhibition of palmitoylation is feasible. An orthogonal approach is to enhance TRIM16 activity or mimic its recognition of ORF3a to accelerate viral protein clearance. Furthermore, combining host‐directed strategies with direct‐acting antivirals or immunomodulators could yield synergistic benefits by simultaneously reducing viral burden and tempering hyperinflammation.

A key methodological feature of this study is the use of a chimeric VSV‐ORF3a virus as a reductionist model to interrogate ORF3a function within a live‐virus context. This system offers several advantages: it enables the isolated study of a single SARS‐CoV‐2 accessory protein within a well‐characterized viral backbone, thereby facilitating mechanistic dissection; it can be handled at lower biosafety levels than authentic SARS‐CoV‐2, which permits higher‐throughput experimentation and in vivo studies; and it provides robust, quantifiable readouts of viral replication and host responses that are readily amenable to genetic and pharmacologic perturbation. However, the VSV‐ORF3a model also has inherent limitations [28, 44]. Its VSV backbone differs fundamentally from SARS‐CoV‐2 in genome organization, replication cycle, cellular and tissue tropism, and the composition of viral and host interaction networks. As such, it cannot fully recapitulate the complexity of SARS‐CoV‐2 infection, and findings from this model should not be interpreted as evidence that VSV and SARS‐CoV‐2 are interchangeable. In this work, we therefore primarily employ VSV‐ORF3a as a discovery and hypothesis‐generating platform, and we explicitly rely on authentic SARS‐CoV‐2 infection models to validate which aspects of the ORF3a palmitoylation–stability axis are conserved in the native coronavirus context.

Several limitations and alternative interpretations warrant consideration. ZDHHC18 appears to be the principal acyltransferase in lung‐relevant cells, redundancy among ZDHHC family members in other tissues or under different inflammatory states remains possible. This possibility is underscored by our observation that ORF3a retains partial palmitoylation even in the absence of ZDHHC18. This residual signal suggests that one or more other ZDHHC enzymes, potentially those with similar activity or localization, may act as a backup pathway with lower efficiency, a common feature of functional redundancy within large enzyme families. Therefore, to fully understand the robustness of this post‐translational regulatory layer, future work should include systematic screening, perhaps utilizing loss‐of‐function approaches or substrate‐trapping mutants, to definitively identify other palmitoyl transferases that may possess minor or context‐dependent catalytic activity toward ORF3a. Such compensatory upregulation could influence the long‐term efficacy of ZDHHC18‐targeted therapies. Moreover, the therapeutic translation of targeting ORF3a palmitoylation require careful evaluation of several factors. These include the off‐target effects of broad palmitoylation inhibitors such as 2‐BP, as well as the pharmacokinetic and immunogenic properties of peptide‐based inhibitors like OPIP. Assessing inhibitor specificity across the ZDHHC family and evaluating potential impacts on essential host palmitoylation pathways will be particularly important. Finally, while TRIM16 is the dominant E3 ligase identified here, other ubiquitin machinery components or deubiquitinases may modulate ORF3a turnover in specific cellular contexts. The role of K27‐linked ubiquitin signaling, which can have non‐proteolytic functions, also merits further exploration. Additionally, given the established role of ORF3a in modulating autophagy, a key future direction is to investigate whether and how its palmitoylation status influences this pathway, which could represent a parallel or interconnected mechanism contributing to viral pathogenesis.

In summary, our integrated molecular, cellular, and in vivo evidence indicates that ZDHHC18‐mediated palmitoylation of ORF3a is a major determinant of its stability, ubiquitination state, and pathogenic potential. By preventing TRIM16‐dependent K27‐linked ubiquitination and proteasomal degradation, palmitoylation preserves ORF3a levels and promotes viral fitness and inflammation. Targeting this palmitoylation‐stability axis, through ZDHHC18 inhibition, substrate mimetic peptides, or modulation of TRIM16, represents a promising host‐directed approach to mitigate ORF3a driven contributions to COVID 19 severity.

## 4 Methods

### 4.1 Ethics Statement

All animal procedures adhered to the guidelines and policies of the Animal Care and Use Committee of the research units. Approval for animal experiments was granted by the Institutional Animal Welfare Committee of Guangzhou National Laboratory (#GZLABAUCP‐2022‐10‐A06). Experiments involving infectious recombinant vesicular stomatitis virus (VSV) strains under Animal Biosafety Level 2 (ABSL2) conditions or SARS‐CoV‐2 EG.5 mutants under ABSL3 conditions were approved by the Institutional Biosafety Committee (IBC) of Guangzhou National Laboratory.

### 4.2 Mice, Cells, and Viruses

8‐weeks‐old female wild‐type Balb/c mice were obtained from SPF GemPharmatech Co., Ltd (Jiangsu, China). K18‐hACE2 transgenic mice (Jax strain 034860) were acquired from Jackson Laboratory via iBio Logistics Co., Ltd (Beijing, China). All mice were housed in a specific pathogen‐free facility under controlled conditions: temperature maintained at 20°C–26°C, relative humidity at 40%–70%, and a 12‐h light/dark cycle.

HEK293 (#CRL‐1573), 293T (#CRL‐3216), HeLa (#CCL‐2), Vero‐E6 (#CRL‐1586), Hepa 1–6 (#CRL‐1830), Huh7, and A549 (#CCL‐185) cells were obtained from ATCC and cultured in Dulbecco's Modified Eagle's Medium (DMEM; Corning #10‐013‐CVR) supplemented with 10% fetal bovine serum (FBS; ExCell Bio #FSP500) and 1% penicillin‐streptomycin (Gibco #15140122). All cell lines were confirmed mycoplasma‐free and maintained at 37°C in a 5% CO_2_ atmosphere.

The SARS‐CoV‐2 EG.5 variant was isolated by the Center for Disease Control and Prevention of Guangdong Province. To construct recombinant VSV carrying SARS‐CoV‐2 ORF3a WT or C130/133S, the coding sequences were inserted into the VSV backbone between the VSV glycoprotein (G protein) and polymerase protein (L protein) using NheI (Thermo Fisher #ER0972) and XhoI (Thermo Fisher #ER0691) restriction enzyme sites. The recombinant viruses (VSV or VSV‐ORF3a WT, VSV‐ORF3a C130/133S) were recovered in Vero‐E6 cells.

To prepare adenoviral shRNA vectors, oligonucleotides targeting mouse Zdhhc18 were designed (target sequence: CCT GAC AAC TAA CGA AGA TAT) and verified to have no homology with other mouse coding genes by BLAST analysis. A 9‐base pair loop sequence (TTC AAG ACG) was inserted between the sense and antisense strands to form a hairpin structure. The shRNA oligos were synthesized by Sangon Biotech (Shanghai, China). The shRNA expression cassette was inserted into the pAdTrack vector between the HindIII and XbaI sites. The recombinant plasmid was linearized and co‐electroporated into E. coli BJ5183 cells with the adenoviral backbone plasmid pAdEasy‐1 for homologous recombination. Positive clones were selected with Ampicillin and confirmed by restriction analysis. The verified recombinant plasmid was then linearized and transfected into HEK293 cells to generate recombinant adenoviruses. A recombinant adenovirus expressing shRNA against Zdhhc18 (Ad5‐Zdhhc18‐shRNA) was generated. A control adenovirus (Ad5‐Control‐shRNA), expressing a non‐targeting scrambled sequence (CTA GGT GTT CTA GTC TGG ACT), was constructed using the same method. All adenoviruses were propagated in HEK293 cells and purified via cesium chloride (CsCl) gradient ultracentrifugation. Total viral particle titers were determined by spectrophotometry at 260 nm, and functional titers were assessed via plaque assays in HEK293 cells.

### 4.3 Antibodies, Reagents, and Peptides

The following antibodies were employed for immunoprecipitation, immunoblotting, and immunofluorescence: mouse anti‐Flag tag monoclonal antibody (biolinkedin #L‐MAb03, 1:3,000); mouse anti‐HA tag monoclonal antibody (Sinobiological #100028‐MM10, 1:3,000]; rabbit anti‐Myc tag polyclonal antibody (proteintech #16286‐1‐AP, 1:3,000); Alpha Tubulin Monoclonal antibody (proteintech #66031‐1‐Ig, 1:3,000); GAPDH Monoclonal antibody (proteintech #60004‐1‐Ig, 1:3,000); Anti‐β‐Actin (ACTB) Antibody (sigma #A1978, 1:3,000); ZDHHC18 polyclonal Antibody (Novus #NBP2‐94111, 1:1,000); ZDHHC6 polyclonal Antibody (Novus # NBP2‐94489, 1:1,000); TRIM16 Polyclonal antibody (proteintech #24403‐1‐AP, 1:2,000); SARS‐CoV2 ORF3a antibody (abcam #ab280953, 1:1000); Streptavidin‐HRP antibody (Thermo Fisher Scientific #21130, 1:5,000); Goat Anti‐Mouse IgG H&L (HRP) (Zenbio #511103, 1:4,000); Goat Anti‐Rabbit IgG H&L (HRP) (Zenbio #511203, 1:4,000); Donkey anti‐Mouse IgG(H+L) Highly Cross‐Adsorbed Secondary Antibody, Alexa Fluor 555 (Invitrogen #A31570, 1:1,000).

2‐bromopalmitate (2BP) (Sigma #18263‐25‐7); Cycloheximide (Selleck #S7418); Hydroxylamine hydrochloride (Sigma #431362); Biotin‐BMCC (Thermo Fisher Scientific #21900); Doxycycline (Dox) (Selleck # S5159); N‐Ethylmaleimide (NEM) (Sigma #E3876); Puromycin (Puro) (Sigma # P9620); IntestiCult Organoid Growth Medium (Mouse) (StemCell Technologies #06000); Matrigel (Corning # 356231); Propidium iodide (PI) (MCE #HY‐D0815); Opti‐MEM (Gibco #31985‐070); Anti‐DYKDDDDK(Flag) magnetic beads (Selleck #B26102); 4% paraformaldehyde (Meilunbio #MA0192‐1); NP‐40 lysis buffer (Beyotime Biotechnology # P0013F); Polybrene (Sigma #H9268); MG132 (Selleck #S2619); 3‐MA (Selleck #S2767); NH_4_Cl (Aladdin Chemistry #A116373); Bortezomib (BTZ) (Selleck # S1013); Chloroquine (CQ) (Selleck # S6999); 15‐Azido‐pentadecanoic acid (MedChemExpress #HY‐151656); DBCO‐PEG4‐Biotin (MedChemExpress #HY‐130809); streptavidin agarose beads (Thermo Fisher Scientific #20349); and β‐mercaptoethanol (Bio‐Rad Laboratories #1610710).

The ORF3a interfering peptide were as follows: OPIP, MRCWLCWKCKSKNPgrkkrrqrrrppq; CTP, MRCWLSWKSKSKNPgrkkrrqrrrppq. Lowercase font indicates the HIV‐TAT sequence. The interfering peptides were synthesized by Sangon at >99% purity and stored at −20°C in powder aliquots of 5 mg. The peptides were dissolved in PBS when using. For the in vivo experiments, the peptides were injected into mice via i.p.

### 4.4 Plasmids

Eukaryotic expression plasmids encoding SARS‐CoV‐2 ORF3a‐Flag/Myc have been described previously, the gene sequence was derived from Wuhan‐Hu‐1(*8*). Sequences for ZDHHCs (ZDHHC2, ZDHHC3, ZDHHC4, ZDHHC6, ZDHHC7, ZDHHC8, ZDHHC9, ZDHHC13, ZDHHC16, ZDHHC18, and ZDHHC20) were amplified from a cDNA library and cloned into pCMV‐3×FLAG/Myc/HA or pcDNA3.1 vectors. Point mutations were introduced via site‐directed mutagenesis using KOD‐Plus polymerase (Toyobo #KFX‐101). CRISPR‐mediated knockout plasmids containing single‐guide RNAs (sgRNAs) targeting human ZDHHC6, ZDHHC18, and TRIM16 (sgZDHHC6, 5’‐TGTATCTCCAGTATTGTAAA‐3’; sgZDHHC18, 5’‐CGCAATCGCTTCTACTGCGG‐3’; and sgTRIM16, 5’‐ GTCGGTGTCAGAGGTCAAAG‐3’) were constructed in the lentiCRISPRv2 backbone (Addgene #108100) following standard protocols. For the tetracycline (Tet) inducible expression plasmid, pCW57‐ORF3a was generated by PCR subcloning ORF3a into pCW57‐MCS1‐2A‐MCS2 (Addgene #71782). All plasmids were verified by Sanger sequencing. Transient transfection of plasmids into HEK293T cells was performed using ExFect Transfection Reagent (Vazyme #T101‐01).

### 4.5 CRISPR–Cas9‐Mediated Gene Knockout

For generating ZDHHC18‐knockout cells, lentiviral particles were produced by transfecting HEK293T cells with pLentiCRISPRv2 vectors containing either scrambled control or target‐specific sequences. The medium was replaced 24 h post‐transfection, and virus‐containing supernatant was collected after 72 h. This supernatant was filtered through a 0.45‐µm membrane, then concentrated using a Universal Virus Concentration Kit (Beyotime #C2901S). HEK293T or A549 cells were infected via incubation with the concentrated lentivirus. To select for transduced cells, puromycin (2 µg/mL) was added to the medium 48 h post‐infection for a 72‐h treatment. Cells were subsequently diluted to 20 cells per 10 mL, plated onto 96‐well plates, and expanded. Single‐cell colonies were manually isolated and validated for ZDHHC18 knockout.

### 4.6 Mouse Lung Organoids Experiments

Primary lung organoid cells were isolated from adult C57BL/6 mouse lungs. Lung tissue was minced into small pieces, washed with pre‐cooled wash solution (DMEM containing 1% FBS and 1% penicillin/streptomycin), and transferred to pre‐warmed digestion medium (DMEM supplemented with 160 U/mL collagenase IV (Gibco #17104019)). Tissue fragments were digested for 1.5 h at 37°C with constant shaking, while release of individual cells was periodically monitored by light microscopy. Digestion was terminated by adding pre‐cooled wash solution. Cell pellets were collected via centrifugation at 300 × g, washed with mouse lung organoid complete medium (AIMINGMED #10‐100‐241), and filtered through a 70‐µm cell strainer to obtain single primary lung organoid cells. Isolated cells were resuspended in Matrigel (Corning #356231) at a final concentration of at least 70% Matrigel and seeded. Matrigel droplets were solidified for 15–30 min in a 37°C, 5% CO_2_ incubator, and 600 µL of pre‐warmed mouse lung organoid complete medium was overlaid per well for culture. Medium was refreshed every 2–3 days. After 7–14 days of culture, pre‐cooled basal medium was added, and Matrigel was disrupted by trituration to harvest organoids. Collected organoids were dissociated into single cells using StemPro Accutase (Gibco #A1110501) incubation at 37°C, and passaged at a 1:3–5 split ratio by re‐seeding into fresh Matrigel.

For infection with recombinant VSV, organoids were resuspended in mouse lung organoid complete medium and incubated with virus at an MOI of 0.01 for 1 h at 37°C on a rotator. Cells were washed once with PBS to remove unbound virus, resuspended in Matrigel (60 µL droplets at 5000 cells), and seeded in 24‐well plates. Matrigel was solidified for 30 min at 37°C before overlaying with 500 µL of lung organoid culture medium. Culture supernatant was collected for virus titration after 18 h. Organoids were then lysed in TRIzol for subsequent qRT‐PCR and transcriptomic analysis.

### 4.7 qPCR

Total RNA was extracted from indicated cells or tissues using TRIzol reagent (Invitrogen #10296010) following the manufacturer's protocol. Subsequently, 1 µg of RNA was reverse‐transcribed with PrimeScript RT Master Mix (Takara #RR036A). RT‐qPCR was performed using SYBR Green Pro Taq HS qPCR Kit (Accurate Biology #AG11733) or One Step PrimeScript RT‐PCR Kit (Vazyme #P612‐01). Target gene expression (human/mouse) was normalized to *GAPDH/Gapdh* and quantified via the 2^(−ΔΔCT) method, with results presented as relative expression. Primer sequences are detailed in Table S1.

### 4.8 Immunofluorescence and Confocal Microscopy

HeLa cells grown on glass coverslips were transfected with plasmids as indicated in the relevant figure panels. Cells were washed with PBS, fixed in 4% paraformaldehyde (PFA) for 30 min at room temperature, and permeabilized with 0.5% Triton X‐100. After blocking with 5% BSA containing 0.1% Tween 20, samples were incubated with primary antibodies (as listed in the ‘Antibodies, reagents and peptides’ section), overnight at 4°C, followed by fluorophore‐conjugated secondary antibodies for 1 h at room temperature. Nuclei were counterstained with DAPI (Sigma–Aldrich #D9542). Following three PBS washes, immunofluorescence images were acquired using a Zeiss LSM800 confocal microscope.

### 4.9 Immunoprecipitation (IP)

Whole‐cell lysates were prepared following transfection or ligand stimulation. Lysates were incubated overnight with specific antibodies coupled to Protein A/G beads (Thermo Scientific #20333/20399) or anti‐Flag beads (Selleck #B26102). Beads were washed three times with lysis buffer (50 mm Tris–HCl pH 8.0, 150 mM NaCl, 1% NP‐40, 10% glycerol). Immunoprecipitated complexes were eluted using 2× SDS Loading Buffer (FD Biotechnology #FD003) and resolved by SDS‐PAGE.

### 4.10 Immunoblot Analysis

Cells were washed twice with ice‐cold PBS and lysed in Lysis Buffer containing Halt Protease Inhibitor Cocktail (Thermo Fisher #78429) for 30 min at 4°C. Lysates were centrifuged at 12 000 ×g (4°C, 10 min), and supernatants were mixed with protein loading buffer, then boiled for 10 min. Samples were separated on 8% or 12% SDS‐PAGE gels and transferred to PVDF membranes. Membranes were blocked with 5% skim milk in PBST (PBS + 0.1% Tween‐20) and probed with primary antibodies overnight at 4°C. After three PBST washes, membranes were incubated with HRP‐conjugated anti‐mouse/anti‐rabbit secondary antibodies. Protein bands were visualized by chemiluminescence and quantified using ImageJ software.

### 4.11 Acyl‐Biotin Exchange (ABE) Assay

The ABE assay was performed as previously described [45]. Briefly, HEK293T cells transiently expressing Flag‐tagged ORF3a or ORF3a point mutants were harvested 48 h post‐transfection. A549 cells infected with recombinant viruses (VSV, VSV‐ORF3a WT, or VSV‐ORF3a C130/133S) were harvested 24 h post‐infection. Prior to lysis, NEM was dissolved in 100% ethanol and added to the lysis buffer (50 mm Tris‐HCl pH 7.5, 150 mM NaCl, 1 mm MgCl_2_, 1% NP‐40, 10% glycerol, protease inhibitors) to a final concentration of 50 mm. Cells were suspended in NEM‐containing lysis buffer for 1.5 h at 4°C, and supernatants were incubated with anti‐Flag beads overnight at 4°C. Beads were washed five times with pH 7.5 lysis buffer and three times with pH 7.2 lysis buffer. Subsequently, beads were incubated with fresh HAM‐containing lysis buffer (50 mm Tris‐HCl pH 7.2, 150 mm NaCl, 1 mm MgCl_2_, 1% NP‐40, 10% glycerol, 1 mm HAM, and protease inhibitors) for 1 h at room temperature, followed by four washes with pH 7.2 lysis buffer and three washes with pH 6.2 lysis buffer. Beads were then treated with 5 µm Biotin‐BMCC in pH 6.2 lysis buffer for 1 h at 4°C. Immunoprecipitates were analyzed by western blot using anti‐Flag antibody and streptavidin‐HRP.

An additional ABE assay was conducted following an established protocol [46]. Briefly, samples were lysed using NP‐40 lysis buffer. Then, 500 µL of the protein lysate was reduced with 50 mm TCEP at 55°C for 1 h, followed by alkylation with 50 mm NEM at 4°C overnight. Proteins were subsequently precipitated with a methanol‐chloroform mixture (500 µL methanol: 250 µL chloroform). The pellet was redissolved in 50 µL of 4% SDS and then biotinylated with 4 mm biotin‐HPDP in a 500 µL reaction volume, either in the presence or absence of 1 m HAM, at 4°C. The biotinylation reaction was allowed to proceed for 2 h for endogenous substrates and 1 h for exogenous substrates. After a second cycle of precipitation and redissolution, the mixture was incubated with 30 µL of streptavidin agarose beads at 4°C overnight. The beads were then washed four times with NP‐40 lysis buffer. Bound proteins were eluted by boiling in 50 µL of 2× Laemmli SDS sample buffer containing 5% β‐mercaptoethanol for 5 min, and were subsequently separated by SDS‐PAGE.

### 4.12 Click‐iT Identification of Palmitoylation

The Click‐iT reaction was performed as previously described with minor modifications [47, 48]. Briefly, HEK293T cells transfected for 36 h with plasmids encoding Flag‐tagged wild‐type ORF3a or its point mutants were treated with 50 µm 15‐azidopentadecanoic acid (MedChemExpress, HY‐151656). Cells were then harvested, lysed, and subjected to centrifugation. The supernatants were incubated with 100 µm DBCO‐PEG4‐biotin (MedChemExpress, HY‐130809) at room temperature for 1 h. Proteins were precipitated using methanol‐chloroform and redissolved in 50 µL of 4% SDS. This solution was diluted to 1 mL, followed by incubation with 30 µL of streptavidin agarose beads overnight at 4°C. After four washes with NP‐40 lysis buffer, bound proteins were eluted by boiling in 50 µL of 2×Laemmli SDS sample buffer (Bio‐Rad Laboratories, #1610710) containing 5% β‐mercaptoethanol (Bio‐Rad Laboratories, #1610737) for 5 min. Proteins were then resolved by SDS‐PAGE.

### 4.13 Mouse Experiment

To establish the Ad5‐shNC or Ad5‐shZdhhc18 model, 6‐weeks‐old male Balb/c mice (purchased from GemPharmatech Co., Ltd., Jiangsu, China) were lightly anesthetized with isoflurane and transduced intranasally with 2.5 × 10^8^ focus‐forming units (FFU) of either Ad5‐shNC or Ad5‐shZdhhc18 in 60 µL of DMEM. 5 days post‐transduction, mice were infected via intravenous injection with recombinant VSV (1 × 10^8^ PFU) in 100 µL DMEM. Mice were euthanized at 3 days postinfection (dpi), and lung tissues were collected. For OPIP testing, female transgenic K18‐hACE2 mice (Jax strain 034860) were divided into three groups (*n* = 5 per group): vehicle, OPIP (20 mg/kg, i.p., QD), or CTP (20 mg/kg, i.p., QD). Mice were then inoculated intranasally with 50 µL of sterile DMEM containing 1 × 10^6^ PFU SARS‐CoV‐2 (EG.5). At 3 dpi, mice were euthanized and lung tissues were collected.

### 4.14 Histological Staining

Lungs from control or virus‐infected mice were dissected, fixed in 10% neutral buffered formalin, embedded in paraffin, sectioned at 4‐µm thickness, and routinely stained with hematoxylin and eosin (H&E). Whole‐slide images were digitized using the NanoZoomer S360 platform (Hamamatsu Photonics).

### 4.15 TCID50

Vero‐E6 cells were seeded in 96‐well plates 24 h prior to infection. Viral culture supernatants were serially diluted in virus maintenance medium (DMEM supplemented with 2% FBS) and inoculated onto Vero‐E6 monolayers at 37°C for 1 h. The inoculum was subsequently removed, and cells were washed once with phosphate‐buffered saline (PBS) to eliminate residual virus. Subsequently, 200 µL of pre‐warmed (37°C) virus maintenance medium was added to each well. After 48 h of incubation, viral titers were quantified as the 50% tissue culture infectious dose (TCID_50_) using the Reed‐Muench method.

### 4.16 Statistical Analysis

All values are expressed as mean ± standard deviation (SD). Statistical analyses were performed using two‐tailed unpaired Student's *t*‐tests or one‐way/two‐way ANOVA (as specified in figure legends) in GraphPad Prism 8.0. Experiments included ≥3 biological replicates. Sample sizes (n, number of mice or samples per experimental group) are detailed in figure legends. Statistical significance was defined as *p* ≤ 0.05. No data points or animals were excluded from analyses. Full statistical methods and replication details are provided in the respective figure legends.

## Author Contributions

Conceptualization was carried out by S.‐D. Y., K. L., L.‐H. L., and D. G. Methodology was performed by L.‐S. Z., Q.‐F. D., J.‐C. H., X.‐R. D., X. W., J.‐W. L., Z.‐H. W., H.‐C. L., and X.‐X. L. Investigation was conducted by H. P., S.‐D. Y., Q.‐F. D., L.‐H. L., and Y.‐X. L. Supervision was provided by D. G. and C.‐M. L. The original draft was written by S.‐D. Y. and K. L., while writing – review and editing was carried out by S.‐D. Y., K. L., L.‐H. L., and D. G.

## Conflicts of Interest

The authors declare no conflicts of interests.

## Supporting information




**Supporting File 1**: advs75591‐sup‐0001‐SuppMat.docx.

**Supporting File 2**: advs75591‐sup‐0002‐Tables.xlsx.

## Data Availability

The data that support the findings of this study are available from the corresponding author upon reasonable request.
