# Supplementary material for: ZDHHC18‐Mediated Palmitoylation of ORF3a Promotes SARS‐CoV‐2 Pathogenesis by Antagonizing TRIM16‐Mediated Ubiquitination and Proteasomal Degradation
Source: Adv Sci (Weinh). 2026 May 10;13(43):e75591. doi: 10.1002/advs.75591 (PMC13335985; doi:10.1002/advs.75591)
Supplement: Supplementary file 1 — Supporting File 1: advs75591‐sup‐0001‐SuppMat.docx. [file ADVS-13-e75591-s002.docx]

**Supplementary Figures**

**ZDHHC18-mediated palmitoylation of ORF3a promotes SARS-CoV-2 pathogenesis by antagonizing TRIM16-mediated ubiquitination and proteasomal degradation**

Sidi Yang^1, 2 #^, Kun Li ^3, #^, Lihong Liu^1, 2#^, Linsen Zeng^1, 2^, Qifeng Deng^4^, Jiacheng Huang^2, 3^, Xiaoran Dong ^2^, Xin Wang^1, 2^, Jianwei Liang^2^, Hongchao Liu^1, 2^, Hong Peng^5^, Yuxin Lin^1, 2^, Xiaolu Xie^1^, Yuzhen Ye^1^, Tiefeng Xu^1^, Zhaohuan Wang^1, 2^, Chun-Mei Li^5^ & Deyin Guo^1,2,3 *^

^1^ State Key Laboratory of Respiratory Disease, National Clinical Research Center for Respiratory Disease, Guangzhou Institute of Respiratory Health, the First Affiliated Hospital of Guangzhou Medical University, Guangzhou 510182, China.

^2^ Guangzhou National Laboratory, Guangzhou International Bio-Island, Guangzhou 510005, China.

^3^ Key Laboratory of Tropical Disease Control of Ministry of Education, Institute of Human Virology, Department of Pathogen Biology and Biosecurity, Zhongshan School of Medicine, Sun Yat-sen University, Guangzhou 510080, China.

^4^ MOE Key Laboratory of Gene Function and Regulation, State Key Laboratory of Biocontrol, School of Life Sciences, Sun Yat-sen University, Guangzhou, 510275 Guangdong, PR China.

^5^ Centre for Infection and Immunity (CII), School of Medicine, Shenzhen Campus of Sun Yat-sen University, Shenzhen 518107, P.R. China.

* Correspondence to: Deyin Guo (guo_deyin@gzlab.ac.cn)

^#^ These authors contributed equally to this work.

**Running title:** ORF3a palmitoylation enhances viral pathogenesis

**
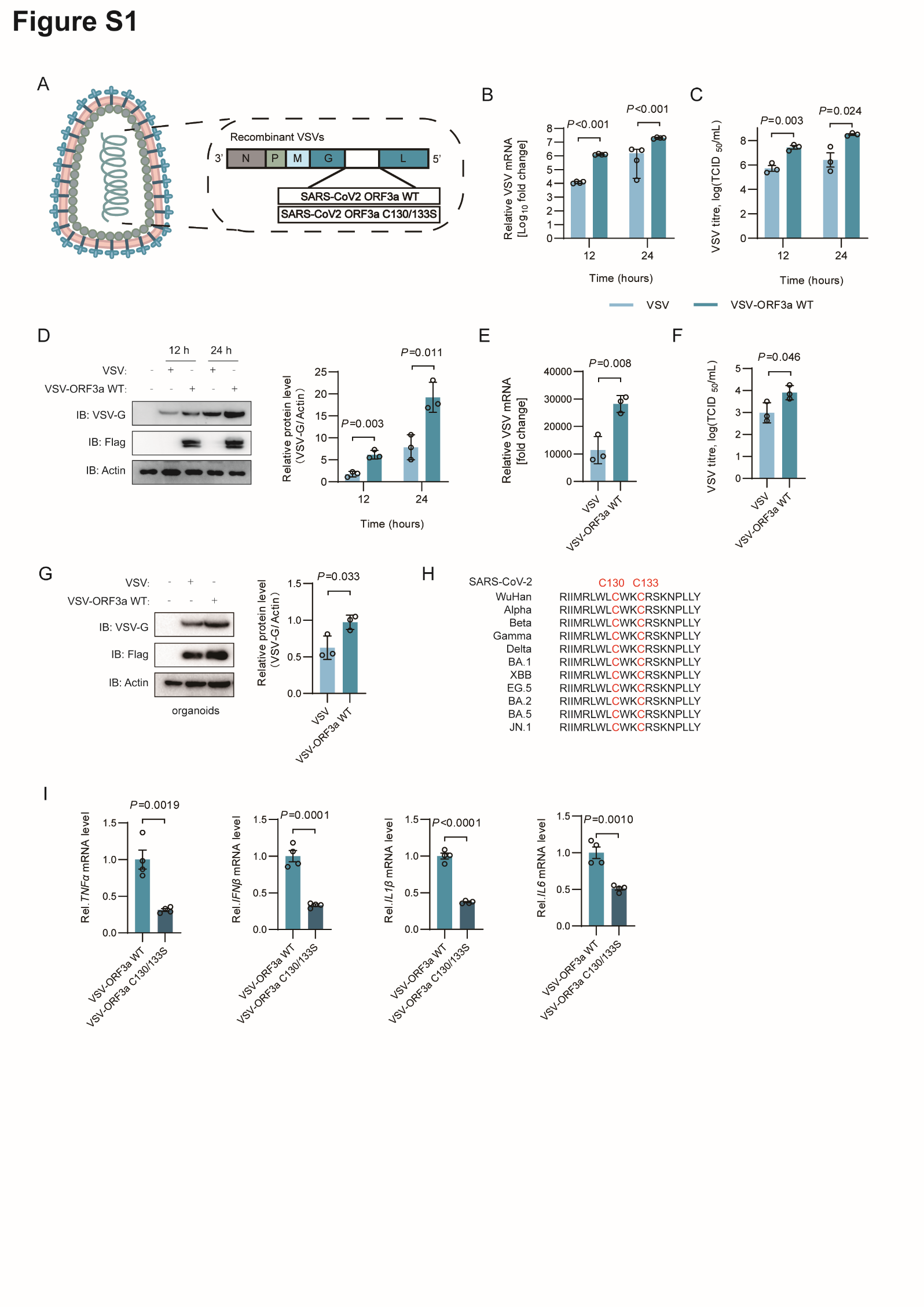
Fig. S1 Recombinant VSV-ORF3a significantly enhanced viral replication. (A)** Schematic diagram of recombinant VSV constructs expressing either wild-type SARS-CoV-2 ORF3a (ORF3a-WT) or the palmitoylation-deficient mutant ORF3a C130/133S. **(B-G)** A549 cells (B-D) and primary lung organoids (E-G) were infected with VSV or VSV-ORF3a WT (MOI = 0.1) for the indicated durations. Fold changes of VSV mRNA (B, E), viral titers (C, F), and VSV-G protein levels (D, G) were quantified at each time point. **(H)** The sequence conservation of ORF3a among SARS-CoV-2 variants was analyzed. **(I)** A549 cells were infected with indicated recombinant VSVs (MOI = 0.1, 16 h) and mRNA levels of cytokines (TNFα, IFNβ, IL1β, IL6) were calculated by qPCR. All data are representative of at least three independent experiments with similar results. Data are presented as Mean ± SD. Statistical significance was determined by unpaired two-tailed Student’s t-test. **
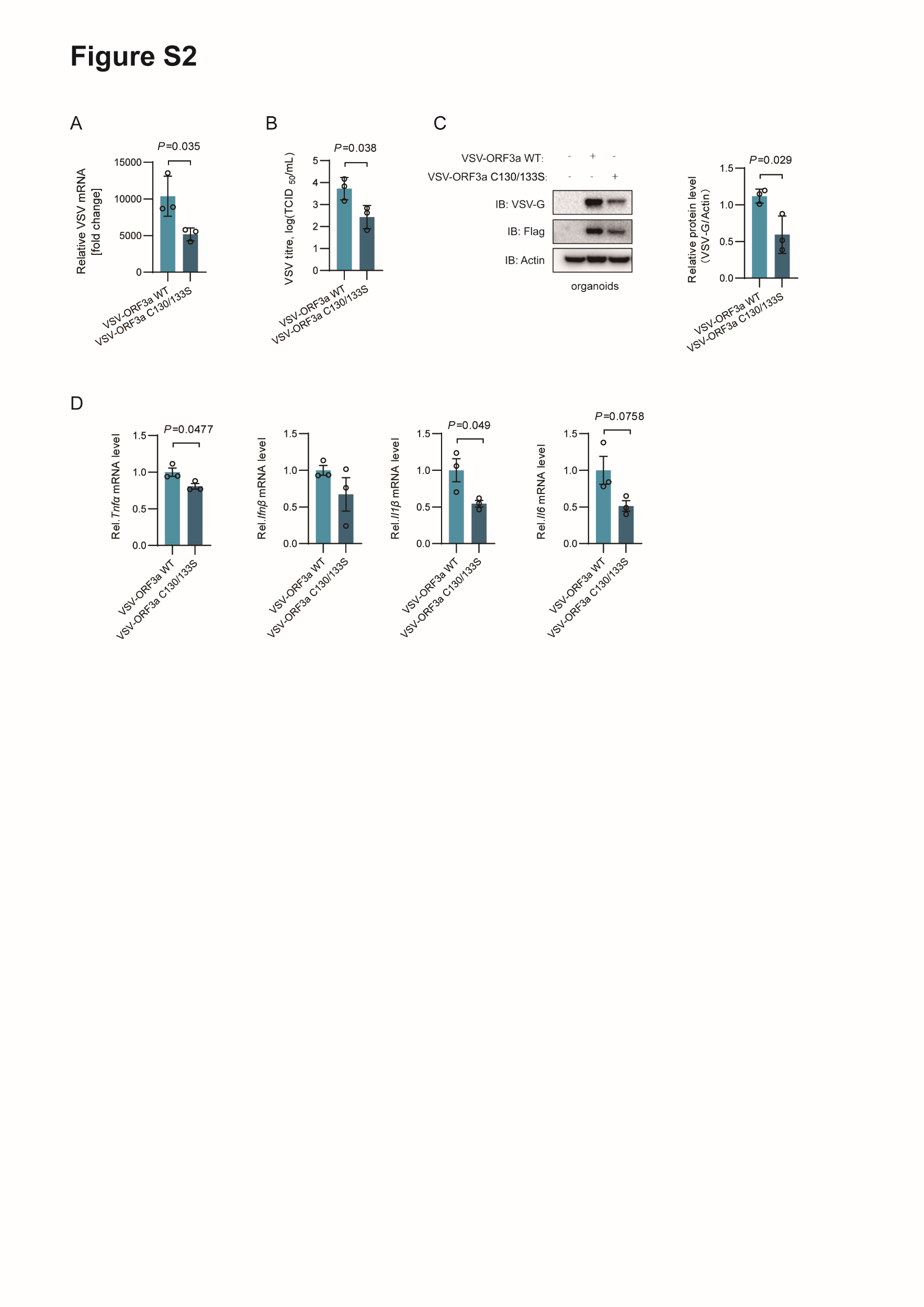
**Abbreviations: IB, immunoblot.

**Fig. S2 Cys130 and Cys133 of ORF3a is essential for viral pathogenesis in lung organoids. (A–C)** Primary lung organoids were infected with VSV-ORF3a WT or VSV-ORF3a C130/133S for 16 h and the fold change of VSV mRNA (A), viral titers (B), and VSV-G protein levels (C) were compared between the two groups. **(D)** The mRNA levels of cytokines (Tnfα, Ifnβ, Il1β, Il6) under identical infection conditions as in (A) were quantified by qPCR. All data are representative of at least three independent experiments with similar results. Data are presented as Mean ± SD. Statistical significance was determined by unpaired two-tailed Student’s t-test. Abbreviations: IB, immunoblot.

**
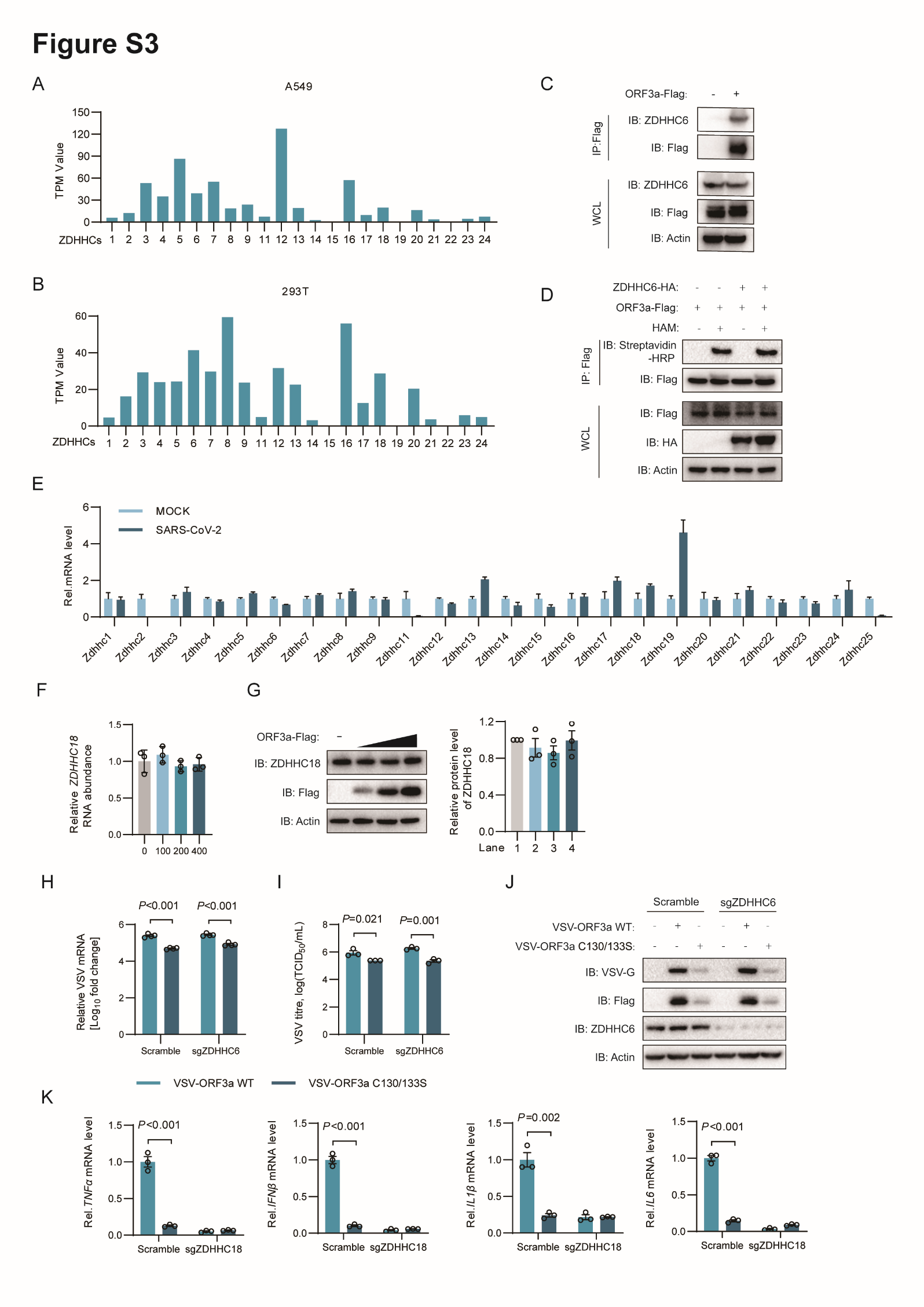
Fig. S3 ZDHHC18 but not ZDHHC6 contributes to ORF3a palmitoylation.** **(A and B)** The expression of different ZDHHCs in (A) A549 cells and (B) HEK293T cells was summarized based on the RNA-seq results of the Human Protein Atlas. **(C)** Endogenous interaction between ORF3a and ZDHHC6 was confirmed by co-immunoprecipitation in HEK293T cells. **(D)** Palmitoylation level of ORF3a was assessed by acyl-biotin exchange (ABE) assay in HEK293T cells transfected with ORF3a-Flag and ZDHHC6-HA. **(E)** K18-hACE2 mice were intranasally inoculated with SARS-CoV-2 (EG.5, 10^6^ PFU per mouse). Zdhhcs mRNA levels were calculated by qPCR in lung homogenates at 3 dpi. **(F, G)** Inducible ORF3a expression HEK293T cells were treated with increasing does of Dox, and fold changes of (F) ZDHHC18 mRNA and (G) ZDHHC18 protein levels were measured. **(H-J)** Scramble control and sgZDHHC6 A549 cells were infected with VSV-ORF3a WT or VSV-ORF3a C130/133S (MOI = 0.1, 16 h). Fold changes of (H) VSV mRNA, (I) viral titers, and (J) VSV-G protein levels were subsequently determined. **(K)** Scrambled control or sgZDHHC18 A549 cells were infected with indicated recombinant VSVs (MOI = 0.1, 16 h) and mRNA levels of cytokines (TNFα, IFNβ, IL1β, IL6) were calculated by qPCR. All data are representative of at least three independent experiments with similar results. Data are presented as Mean ± SD. n = 3 independent samples (F-I, K). Statistical significance was determined by unpaired two-tailed Student’s t-test. Abbreviations: IP, immunoprecipitation; IB, immunoblot; WCL, whole-cell lysates; HAM, hydroxylamine; Dox, doxycycline.

**
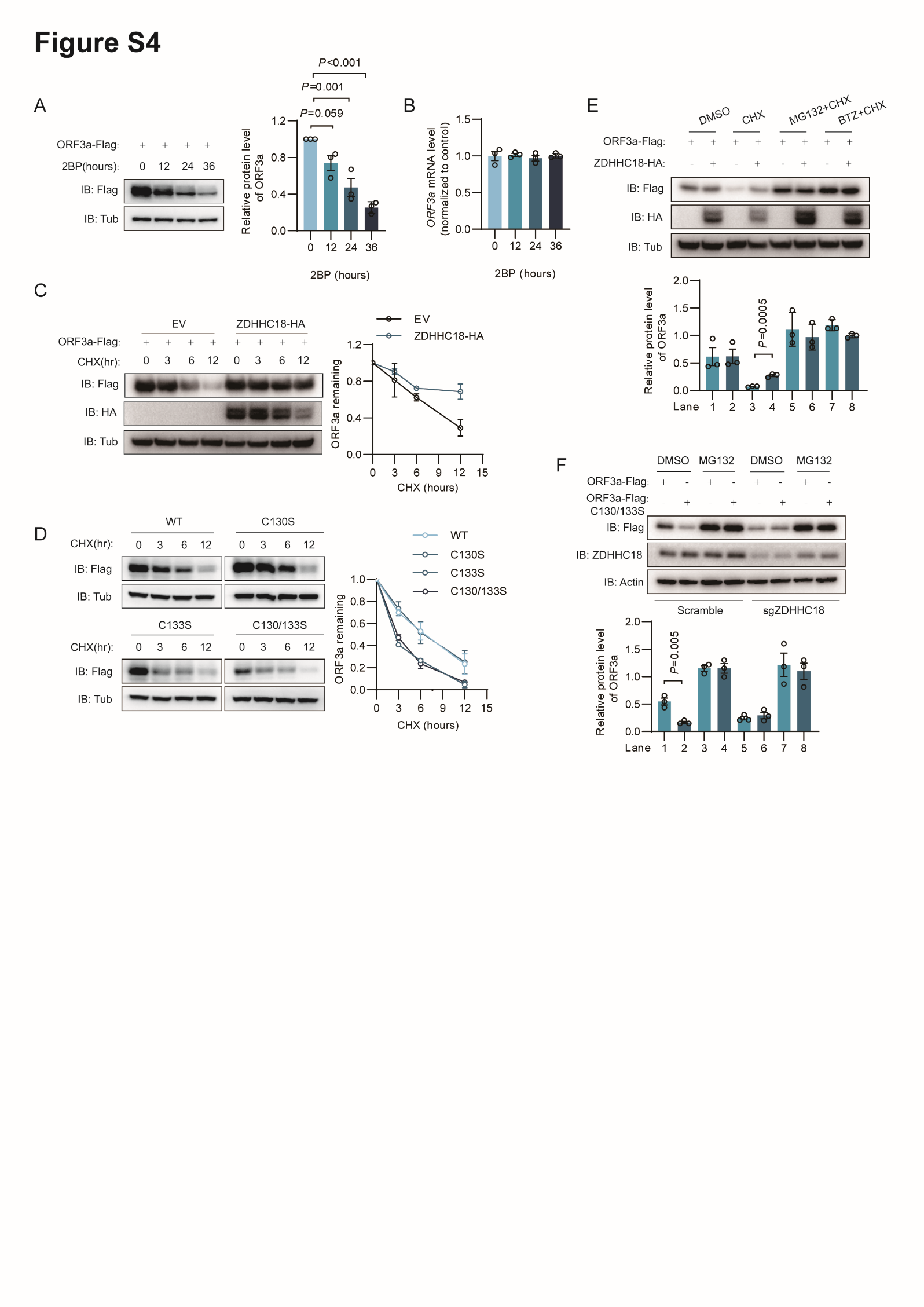
Fig. S4 Palmitoylation of ORF3a mediated by ZDHHC18 inhibits its degradation through proteasome pathway. (A and B)** HEK293T cells were transfected with ORF3a-Flag and treated with 2BP for the indicated durations. Cell lysates were collected for (A) IB and (B) qPCR. **(C)** IB analysis of ORF3a expression in HEK293T cells transfected with ORF3a-Flag and ZDHHC18-HA and treated with CHX at the indicated timepoints. **(D)** IB analysis of ORF3a expression in HEK293T cells transfected with ORF3a-Flag, ORF3a-Flag C130S, ORF3a-Flag C133S or the double mutant ORF3a-Flag C130/133S mutant, and subsequently treated with CHX for the indicated time points. **(E)** IB analysis of ORF3a expression in HEK293T cells transfected with ORF3a-Flag and ZDHHC18-HA and then treated with CHX alone or together with the proteasome inhibitors MG132 or BTZ. **(F)** IB analysis of ORF3a expression in scrambled control versus sgZDHHC18 HEK293T cells transfected with ORF3a-Flag or ORF3a-Flag C130/133S mutant and treated with DMSO (as control) or MG132. All data are representative of at least three independent experiments with similar results. Data are presented as Mean ± SD. n = 3 independent samples (A-F). Statistical significance was determined by unpaired two-tailed Student’s t-test or one-way ANOVA. Abbreviations: 2BP, 2-bromopalmitate; IB, immunoblot; BTZ, bortezomib; CHX, cycloheximide.


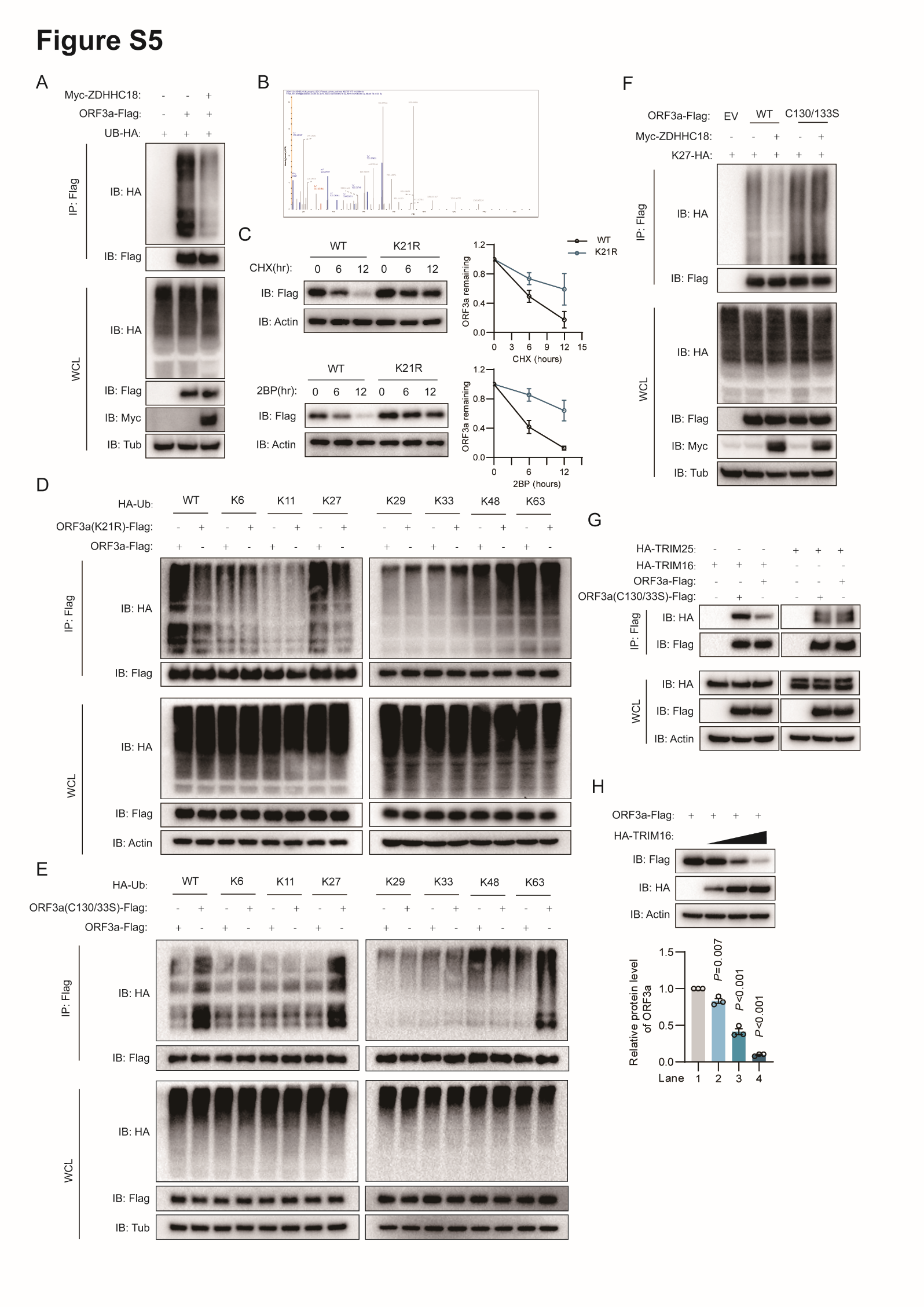
**Fig. S5** **ZDHHC18 competitively inhibits TRIM16-mediated K27-linked polyubiquitination. (A)** IP analysis of ORF3a ubiquitination in HEK293T cells transfected with ORF3a-Flag and Myc-ZDHHC18. Cells were treated with MG-132 for 4 hours before sample collection. **(B)** Mass spectrometry analysis of ORF3a immunoprecipitants to show the primary ubiquitination site. **(C)** IB analysis of ORF3a expression in HEK293T cells transfected with ORF3a-Flag or ORF3a-Flag K21R mutant and then treated with CHX or 2BP for the indicated timepoints. **(D)** IP analysis of ORF3a and ORF3a K21R mutant ubiquitination in HEK293T cells transfected with ORF3a-Flag or ORF3a-Flag K21R mutant along with HA-Ub-WT or its mutants. Cells were treated with MG-132 for 4 hours before sample collection. **(E)** IP analysis of ORF3a and the ORF3a C130/133S mutant ubiquitination in HEK293T cells transfected with ORF3a-Flag or ORF3a-Flag C130/133S mutant along with HA-Ub-WT or its mutants. Cells were treated with MG-132 for 4 hours before sample collection. **(F)** IP analysis of K27-linked ubiquitination of ORF3a and ORF3a C130/133S mutant in HEK293T cells transfected with Myc-ZDHHC18 and either ORF3a-Flag or ORF3a-Flag C130/133S mutant. Cells were treated with MG-132 for 4 hours before sample collection. **(G)** IB analysis of WCL and anti-Flag immunoprecipitant from HEK293T cells transfected with ORF3a-Flag, ORF3a-Flag C130/133S mutant and HA-TRIM16 or HA-TRIM25 as indicated. **(H)** IB analysis of ORF3a expression in HEK293T cells transfected with ORF3a-Flag and increasing doses of HA-TRIM16. All data are representative of at least three independent experiments with similar results. Data are presented as Mean ± SD. n = 3 independent samples (C, H). Statistical significance was determined by one-way ANOVA. Abbreviations: IP, immunoprecipitation; IB, immunoblot; WCL, whole-cell lysates; 2BP, 2-bromopalmitate; CHX, cycloheximide.

**
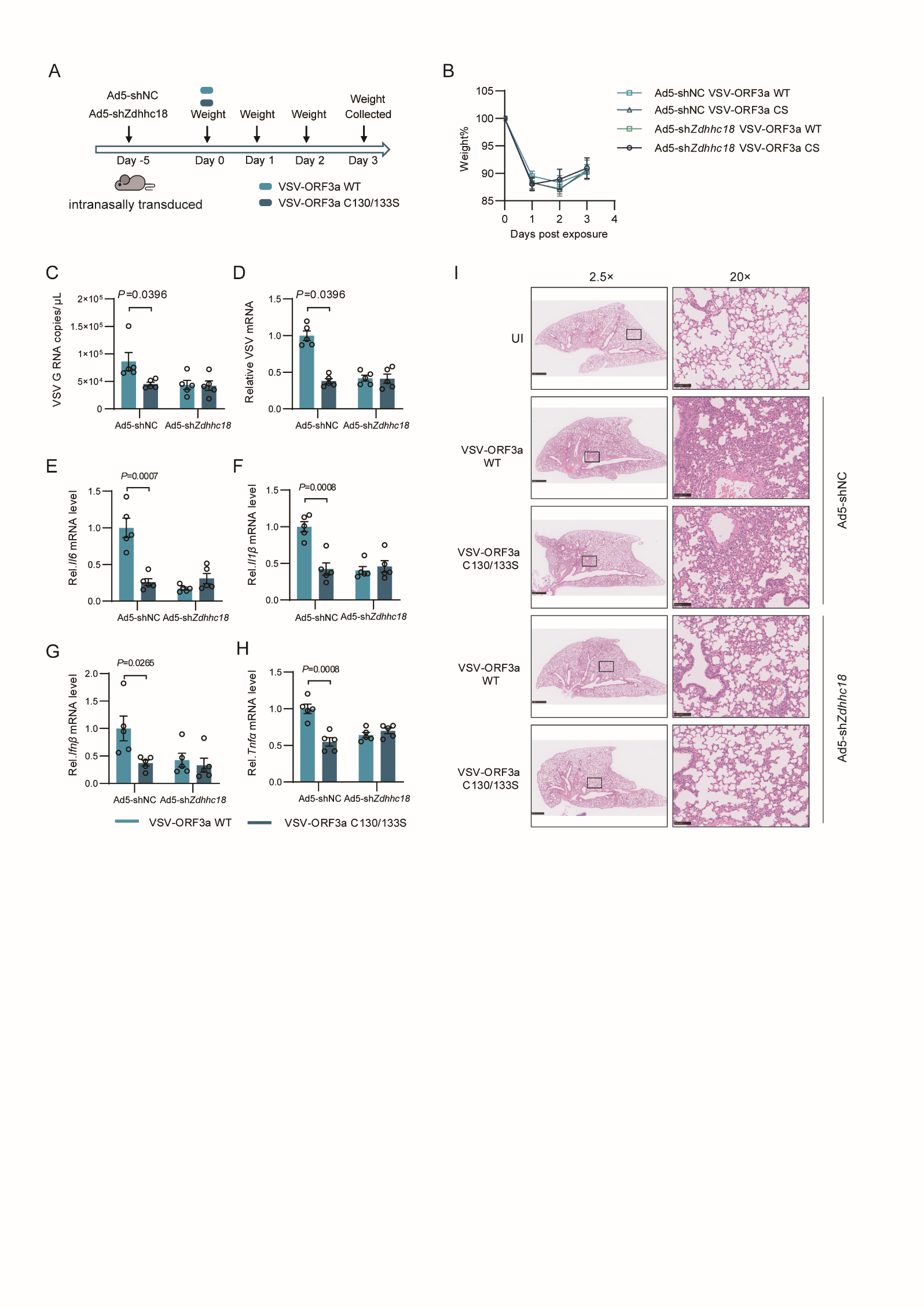
Fig.S6 ZDHHC18-mediated palmitoylation of ORF3a is critical for viral pathogenesis in the VSV-ORF3a chimeric model. (A)** The schematic diagrams of Ad5-sh*Zdhhc18* transduced mice and subsequent recombinant VSVs challenge. **(B-I)** Ad5-Zdhhc18 transduced Balb/c mice were intranasally infected with 1.5 × 10^8^ PFU of VSV-ORF3a WT or VSV-ORF3a C130/133S mutant in 60 μL DMEM. Body Weight changes were monitored daily (B). At 3 days post-infection, viral titers in lungs were measured through VSV genome copy numbers (primer/probes target the G gene) (C) and relative VSV G protein mRNA levels by qPCR (D). Cytokine mRNA levels of Il6 (E), Il1β (F), Ifnβ (G), Tnfα (H) were measured by qPCR. The tissue injury in lung sections was analyzed by hematoxylin and eosin staining I). Scale bar, 1 mm (Left) or 100 µm (Right). Data are presented as Mean ± SD. Statistical significance was determined by unpaired two-tailed Student’s t-test. Abbreviations: IB, immunoblot.

**
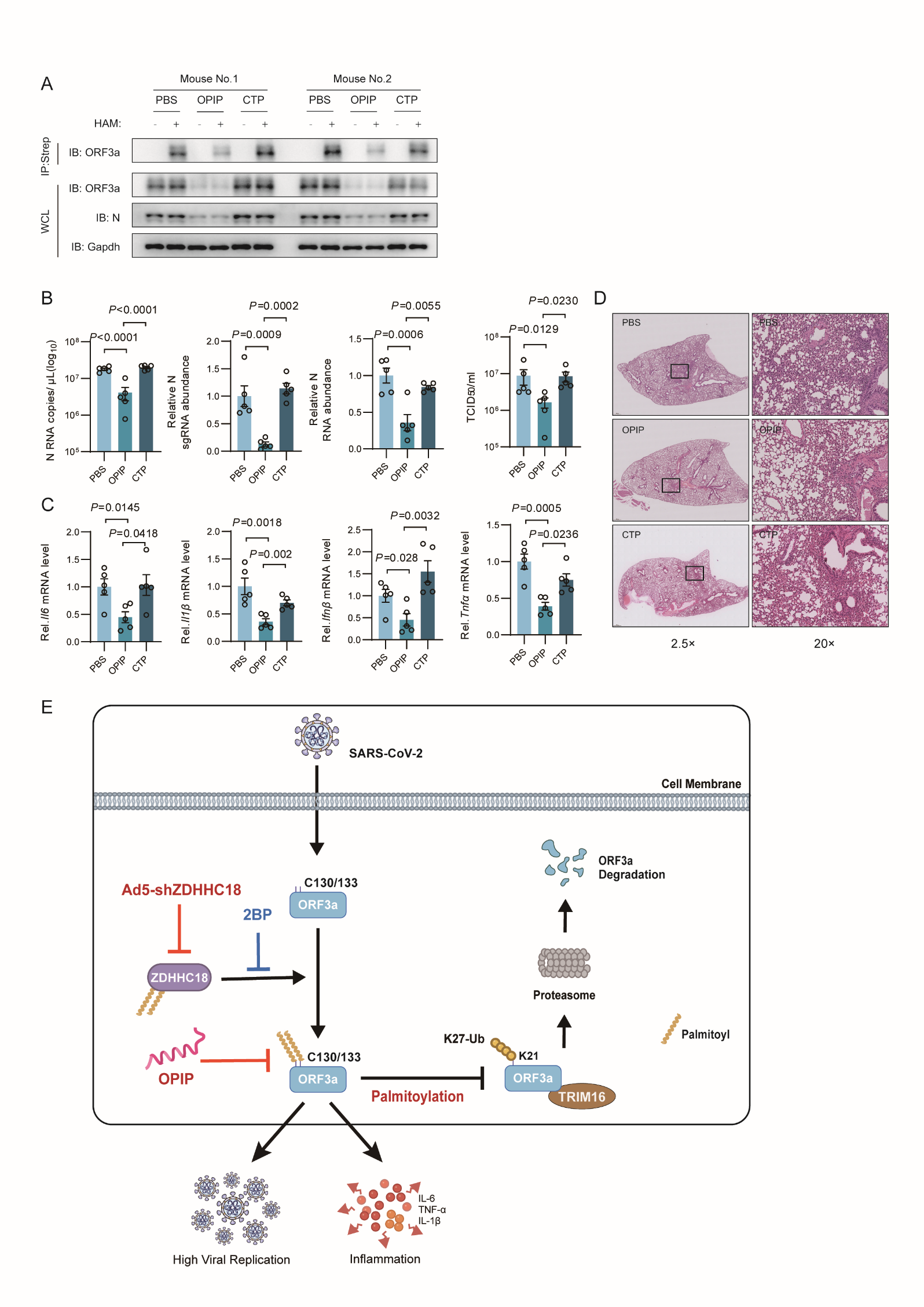
Fig. S7 The peptide OPIP targeting ORF3a palmitoylation limits SARS-CoV-2 infection in vivo.** **(A-D)** K18-hACE2 mice were intranasally inoculated with SARS-CoV-2 (Wuhan-Hu1, 500 PFU per mouse) and treated with PBS, OPIP or CTP (20 mg/kg, i.p., QD). (A) Endogenous ORF3a palmitoylation levels in lung tissues were assessed by ABE assay coupled with immunoblot analysis. (B) SARS-CoV-2 genome copy numbers (targeting the N gene), N sgRNA levels and SARS-CoV-2 titers in lungs were measured at 3 dpi. (C) Cytokine Il6, Il1β, Ifnβ and Tnfα mRNA levels in lung homogenates at 3 dpi were measured by qPCR. (D) The tissue injury in lung sections was analyzed by hematoxylin and eosin staining. Scale bar, 1 mm (Left) or 100 µm (Right). **(E)** Model of ZDHHC18-mediated ORF3a protein palmitoylation enhances SARS-CoV-2 virulence: Palmitoylation by ZDHHC18 blocks ORF3a K27-linked ubiquitination mediated by TRIM16, thereby preventing its proteasomal degradation and strengthening viral pathogenesis. Targeting palmitoylation through a pharmacological inhibitor (2-BP), a competitive inhibitory peptide (OPIP), or adenovirus-mediated knockdown of ZDHHC18 expression presents a potential therapeutic strategy against SARS-CoV-2. This approach may mitigate pathogenesis by reducing ORF3a expression, offering proof-of-concept for a novel host-directed antiviral treatment. All data are representative of at least three independent experiments with similar results. Data are presented as Mean ± SD. Statistical significance was determined by unpaired two-tailed Student’s t-test. Abbreviations: 2BP, 2-bromopalmitate.
